# Learning Subject-Specific Directed Acyclic Graphs With Mixed Effects Structural Equation Models From Observational Data

**DOI:** 10.3389/fgene.2018.00430

**Published:** 2018-10-02

**Authors:** Xiang Li, Shanghong Xie, Peter McColgan, Sarah J. Tabrizi, Rachael I. Scahill, Donglin Zeng, Yuanjia Wang

**Affiliations:** ^1^Statistics and Decision Sciences, Janssen Research and Development, LLC, Raritan, NJ, United States; ^2^Department of Biostatistics, Mailman School of Public Health, Columbia University, New York, NY, United States; ^3^National Hospital for Neurology and Neurosurgery, London, United Kingdom; ^4^Department of Biostatistics, University of North Carolina, Chapel Hill, NC, United States; ^5^Departments of Psychiatry, Columbia University Medical Center, New York, NY, United States

**Keywords:** graphical models, network analysis, causal structure discovery, heterogeneity, regularization

## Abstract

The identification of causal relationships between random variables from large-scale observational data using directed acyclic graphs (DAG) is highly challenging. We propose a new mixed-effects structural equation model (mSEM) framework to estimate subject-specific DAGs, where we represent joint distribution of random variables in the DAG as a set of structural causal equations with mixed effects. The directed edges between nodes depend on observed exogenous covariates on each of the individual and unobserved latent variables. The strength of the connection is decomposed into a fixed-effect term representing the average causal effect given the covariates and a random effect term representing the latent causal effect due to unobserved pathways. The advantage of such decomposition is to capture essential asymmetric structural information and heterogeneity between DAGs in order to allow for the identification of causal structure with observational data. In addition, by pooling information across subject-specific DAGs, we can identify causal structure with a high probability and estimate subject-specific networks with a high precision. We propose a penalized likelihood-based approach to handle multi-dimensionality of the DAG model. We propose a fast, iterative computational algorithm, DAG-MM, to estimate parameters in mSEM and achieve desirable sparsity by hard-thresholding the edges. We theoretically prove the identifiability of mSEM. Using simulations and an application to protein signaling data, we show substantially improved performances when compared to existing methods and consistent results with a network estimated from interventional data. Lastly, we identify gray matter atrophy networks in regions of brain from patients with Huntington's disease and corroborate our findings using white matter connectivity data collected from an independent study.

## 1. Introduction

Directed acyclic graphs (DAGs) are used to represent the causal mechanisms of a complex system of interacting components, such as biological cellular pathways (Sachs et al., 2005), gene regulatory networks (Ud-Dean et al., 2016), and brain connectivity networks (Friston, 2011). The ability to identify causal relations between variables in observational data is highly challenging. Specifically, given a set of centered random variables $\text{M}={\left(M_{1},\cdots \;,M_{p}\right)}^{\prime }$, referred to as nodes, the causal relationship between these nodes in a DAG can be represented by a structural equation model (SEM) (Pearl, 2009):

$$M_{j}=f_{j}\left(\text{pa}\left(j\right),\varepsilon_{j}\right),\;j=1,\cdots \;,p,$$

where pa(*j*) is the set of parental nodes of *M*_*j*_, and ε_*j*_ is a random variable representing unexplained variation. In many applications, **M** is assumed to follow a multivariate Gaussian distribution satisfying a linear SEM,

(1) $$\begin{array}{r} M_{j}=\underset{k\in \text{pa}\left(j\right)}{\sum }\theta_{jk}M_{k}+\varepsilon_{j},\;\varepsilon_{j}\sim N\left(0,\sigma_{j}^{2}\right);\;j=1,\cdots \;,p, \end{array}$$

where **B** = (θ_*jk*_) is referred to as an adjacency matrix.

Estimation of DAG structure (i.e., parental sets pa(*j*)) is non-deterministic polynomial-time hard (NP-hard) because the number of possible DAGs grows super-exponentially with the number of nodes (Robinson, 1971). Mainly two types of methods are proposed to tackle this challenge, namely, independence-based (e.g., Spirtes et al., 2000) and score-based (e.g., Heckerman et al., 1995) methods. The independence-based approaches calculate the partial correlation between any pair of nodes and perform statistical tests to assess the conditional dependence. A popular method is the PC algorithm (Spirtes et al., 2000), which has been proven to be uniformly consistent for estimating ultra high-dimensional, sparse DAGs (Kalisch and Bühlmann, 2007). The PC algorithm was modified as PC-stable to remove its dependence on node ordering (Colombo and Maathuis, 2014). A limitation of the PC algorithm is that it does not provide the proper level of multiple comparison correction and thus may lead to a large number of false positives in practice. To remedy this limitation, a hybrid, two-stage approach was proposed (PenPC, Ha et al., 2016) that first estimates a sparse skeleton based on penalized regression and then performs a modified PC-stable algorithm on the skeleton.

The score-based approach searches for the DAG using a pre-specified score criterion, such as Bayesian Information Criterion (BIC) or penalized likelihood function. As it is not computationally feasible to search through the space of all DAGs, a two-phase greedy equivalence search algorithm explores an equivalence class based on BIC by adding and deleting edges. With additional information on node ordering, the estimation of DAG is equivalent to neighborhood selection for which several penalized likelihood approaches have been developed (Shojaie and Michailidis, 2010; Yuan et al., 2012). More recently, attempts have been made to estimate a DAG without knowing the node ordering (Aragam and Zhou, 2015; Han et al., 2016). Other recent developments include leveraging asymmetric information between nodes (Shimizu et al., 2006; Luo and Zhao, 2011) or exploring the invariance property of causal relation using combined observational and interventional data (Meinshausen et al., 2016). Simulation studies suggest that independence-based methods perform adequately for identifying the skeleton of a DAG from observational data (Smith et al., 2011). However, these methods may perform worse for identifying the causal direction than some search-and-score methods that exploit the asymmetric distributional information (Smith et al., 2011).

All of the existing DAG estimation methods assume homogeneity of the causal effect of the underlying DAG model in (1) (i.e., θ_*jk*_ is common across individuals in the population). However, there is a growing body of evidence suggesting that biological networks may depend on subject-specific characteristics such as genomic markers (Brown et al., 2011; Bohlken et al., 2016; Langfelder et al., 2016). For mental disorders, individual differences in edge strength in comorbidity networks have been widely observed (Fleeson et al., 2010). Modeling heterogeneity of network effects may improve interpretability, biological relevance, and predictability. This area is much less explored with the exception of a few methods proposed to study subject-specific undirected graphical models. For example, a conditional Gaussian graphical model with covariate-adjusted mean but homogeneous precision matrix has been considered (Yin and Li, 2011; Cai et al., 2013). To characterize heterogeneous dependence structure between groups, Guo et al. (2015) jointly estimated graphical models that share common structure but also allowed for differences between networks. Recently, instead of modeling groups separately, Cheng et al. (2014) directly incorporated covariates into an Ising model in order to build a covariate-dependent undirected graph. A common assumption of these approaches is that the dependence between two nodes is fully explained by the observed exogenous covariates. Such an assumption may not be satisfied in many biological and clinical applications due to the presence of unexplained latent residual heterogeneity representing hidden pathways between nodes. Shimizu and Bollen (2014) proposed a Bayesian approach to estimate DAG by including non-Gaussian latent variables in a linear SEM, but does not estimate individualspecific graphs

Our goal in this article is to develop a novel method and an efficient estimation procedure to study covariate-dependent DAGs with latent effect modification in multi-dimensional settings. Our method is based on mixed-effects SEM (mSEM) and penalized likelihood to obtain DAG structure and causal effects simultaneously. The covariates are treated as exogenous variables, and their joint distribution is not of interest. The key difference between mSEM and current approaches is that the causal effect, θ_*jk*_ in Model (1), is random and varies across individuals. To capture variation of the manifestation of causal relationship among individuals, our model allows the magnitude of the edge strength to be heterogeneous across subjects, while keeping the direction of causal relationship to be homogeneous. The heterogeneous causal magnitude is modeled by both fixed effects that depend on observed covariates and random effects that capture unexplained heterogeneity.

We propose a two-stage approach to estimate mSEM, whereby the first stage performs neighborhood selection by maximizing a penalized likelihood to identify a sparse skeleton, and the second stage searches for the DAG by solving an approximate ℓ_0_-penalty problem via hard-thresholding within the identified skeleton, followed by an easily implemented DAG-checking procedure. We show theoretical proof of the identifiability (the graph is unique) of our model. Through extensive simulations and application to a well-known protein signaling study (Sachs et al., 2005), we show substantially improved performance in terms of robustness and accuracy when compared to existing methods, including PC (Spirtes et al., 2000) and penPC (Ha et al., 2016), and consistent performance when compared to analysis using interventional data. Lastly, we apply the proposed method to discover the causal dependence relationship among regions of brain atrophy from patients with Huntington's disease (HD) (Paulsen et al., 2014) and corroborate our findings in an independent study (McColgan et al., 2017).

## 2. Methodology

For the *i*th subject, let ${\text{M}}_{i}={\left(M_{i1},M_{i2},\cdots \;,M_{ip}\right)}^{\prime }$ denote *p* random variables or nodes in a DAG. Let ${\text{X}}_{i}={\left(1,X_{i1},X_{i2},\cdots \;,X_{iq}\right)}^{\prime }$ denote a *q*+1-dimensional vector including a constant and *q* exogenous covariates that may modify the causal network among components in **M**_*i*_. We consider a mixed-effects model in which the causal effect depends on both fixed effects of observed variables **X**_*i*_ and unobserved random effects {γ_*ijk*_}. For the *j*th node, the mSEM is given by:

(2) $$\begin{array}{r} M_{ij}=\underset{k\in \text{pa}\left(j\right)}{\sum }\left(\beta_{jk}^{T}{\text{X}}_{i}+\gamma_{ijk}\right)M_{ik}+\varepsilon_{ij}, \end{array}$$

where β_*jk*_ is the vector of fixed effects (including an intercept and effects associated with **X**_*i*_), and γ_*ijk*_ is the unexplained heterogeneity of causal effects beyond **X**_*i*_. We assume that γ_*ijk*_ are independent and follow $N\left(0,\sigma_{jk}^{2}\right)$ and the independent error terms ε_*ij*_ follow $N\left(0,\sigma_{\varepsilon_{j}}^{2}\right)$. The SEM in (2) assumes that for each edge in the DAG, the causal effect is decomposed into a subject-specific fixed-effect term that depends on the exogenous covariates (i.e., $\beta_{jk}^{T}{\text{X}}_{i}$) and a subject-specific random-effect term that captures residual heterogeneity in causal effects due to other latent factors beyond **X**_*i*_ (i.e., γ_*ijk*_). When β_*jk*_ = 0 and $\sigma_{jk}^{2}\neq 0$, the causal dependence between *j* and *k* is explained by unobserved latent factors but not **X**_*i*_. No causal effect between node *j* and *k* corresponds to β_*jk*_ = 0 and $\sigma_{jk}^{2}=0$.

In this work, we assume that the ordering of causal dependence or the parental sets are unknown, and propose methods to simultaneously learn the ordering and structure of DAG and the parameters in the SEM. Previous literature has pointed out that qualitative capacity claims about causal effects are invariant across different populations of subjects, whereas the quantitative claims in SEM often are population-specific (e.g., Woodward, 2005, Chapter 7). Thus, we assume that the qualitative causal dependence (set of nodes and directed edges) is homogeneous among subjects while the magnitude of the edge strength varies across subjects. Presence of an edge from *M*_*ik*_ to *M*_*ij*_ is defined as β_*jk*_ ≠ **0** or $\sigma_{jk}^{2}\neq 0$; otherwise, there is no causal effect from *M*_*ik*_ to *M*_*ij*_. Note that when the components of β_*jk*_ associated with covariates *X*_*il*_ are zero and $\sigma_{jk}^{2}$ are zero, the subject-specific DAG model in (2) reduces to a homogeneous DAG model in (1). We express the model for **M**_*i*_ given γ_*ijk*_ in matrix form as

(3) $${\text{M}}_{i}=(\text{B}({\text{X}}_{i})+\Gamma_{i}){\text{M}}_{i}+\varepsilon_{i}$$

where **B**(**X**_*i*_) is a matrix of fixed effects with entry (*j, k*) as $\beta_{jk}^{T}{\text{X}}_{i}$ and the diagonal elements as zeros, Γ_*i*_ is a matrix of random effects with entry (*j, k*) as γ_*ijk*_ and the diagonal elements as zeros, and $\varepsilon_{i}={\left(\varepsilon_{i1},\varepsilon_{i2},\cdots \;,\varepsilon_{ip}\right)}^{\prime }$ is a vector of error terms. Note that the joint distribution of **M** in Model (3) is non-Gaussian due to random effects in Γ_*i*_, where the asymmetric information on the distribution between nodes can facilitate inference on the causal network from the observational data.

To estimate a DAG, we use a likelihood-based approach. Given the random effects Γ_*i*_, the conditional likelihood function of **M**_*i*_ is given by

(4) $$\begin{array}{l} p({\text{M}}_{i};{\text{X}}_{i}|\Gamma_{i})\propto \;|\text{E}{|}^{-1/2}|\text{I}-\text{B}({\text{X}}_{i})-\Gamma_{i}|\times \\ \exp\left(-\frac{1}{2}{\text{M}}_{i}^{T}{\left(\text{I}-\text{B}({\text{X}}_{i})-\Gamma_{i}\right)}^{T}{\text{E}}^{-1}(\text{I}-\text{B}({\text{X}}_{i})-\Gamma_{i}){\text{M}}_{i}\right), \end{array}$$

where Cov[**ε**_*i*_] = **E** is a diagonal matrix of $\sigma_{\varepsilon_{j}}^{2}$. The derivation of (4) is given in the online Supplementary Material Section S1.

To simplify presentation, we introduce the notation for the vectorized Γ_*i*_ and define non-zero components of vectorized Γ_*i*_ as $\gamma_{i}=\left\{\gamma_{ijk}:\sigma_{jk}^{2}>0\right\}$. Then, Γ_*i*_ can be expressed as a linear combination of components in **γ**_*i*_ as $\Gamma_{i}=\underset{\sigma_{jk}^{2}>0}{\sum }\gamma_{ijk}{\text{H}}_{jk},$ where **H**_*jk*_ is a single-entry matrix with one entry (*j, k*). Denote by $\text{Cov}\left[\gamma_{i}\right]=\text{diag}\left\{\sigma_{12}^{2},\ldots ,\sigma_{jk}^{2},\ldots ,\sigma_{pp-1}^{2}\right\}=\text{G}$ the covariance matrix of **γ**_*i*_. The observed likelihood function is given by

(5) $$\begin{array}{r} \overset{n}{\underset{i=1}{\prod }}\underset{\gamma_{i}}{\int }p\left({\text{M}}_{i};{\text{X}}_{i}|\gamma_{i}\right)p\left(\gamma_{i}\right)\text{d}\gamma_{i}, \end{array}$$

where $p\left(\gamma_{i}\right)\propto |\text{G}{|}^{-1/2}\exp\left(-\gamma_{i}^{T}{\text{G}}^{-1}\gamma_{i}/2\right)$.

Under the DAG assumption of no directed cycle, **B**(**X**_*i*_)+Γ_*i*_ can always be transformed into an upper diagonal matrix after some unknown permutation of the rows and columns. Therefore, the determinant |**I**−**B**(**X**_*i*_)−Γ_*i*_| in the likelihood function (4) is one. The integral in the likelihood (5) can be explicitly calculated up to a constant and the negative log-likelihood function is given by

(6) $$\begin{array}{l} l_{n}=\sum_{i=1}^{n}\sum_{j=1}^{p}(\frac{(M_{ij}-\sum_{k\neq j}(\beta_{jk}^{T}{\text{X}}_{i})M_{ik}{)}^{2}}{\sum_{k\neq j}\sigma_{jk}^{2}M_{ik}^{2}+\sigma_{\varepsilon_{j}}^{2}}\\ \;+\log\left(\sum_{k\neq j}\sigma_{jk}^{2}M_{ik}^{2}+\sigma_{\varepsilon_{j}}^{2}\right)), \end{array}$$

where the detailed derivations are given in the online Supplementary Material Section S1. Based on the objective function (6), the parameter estimation for each node in the likelihood is separable, leading to significant computational advantage. Note that for node *j*, the negative log-likelihood function (6) is equivalent to the objective function of a weighted least squares. Therefore, to obtain initial values, one can use weighted least squares to update {β_*jk*_:*j* = 1, ⋯, *p*; *k* = 1, ⋯, *p*} and use the Newton-Raphson algorithm to update $\left\{\sigma_{jk}^{2}:j=1,\cdots \;,p;k=1,\cdots \;,p\right\}$ and $\left\{\sigma_{\varepsilon_{j}}^{2}:j=1,\cdots \;,p\right\}$ until convergence. The identifiability of parameters in the model is shown in Theorem 1 in section 2.3.

### 2.1. Initial sparse graph

With a large number of nodes, minimizing (6) would result in a full graph with all non-null estimates of {_*jk*_} and $\sigma_{jk}^{2}$. Without any constraint on the estimates, the graph may potentially involve many false positive edges. To accommodate the large number of nodes, we propose to use a penalized likelihood to choose an initial sparse graph skeleton and search for the optimum of (6) within this reduced graph space. Based on model (2), the marginal expectation and variance of *M*_*ij*_ are $\sum_{k\neq j}(\beta_{jk}^{T}{\text{X}}_{i})M_{ik}$ and $\sigma_{\varepsilon_{j}}^{2}+\sum_{k\neq j}\sigma_{jk}^{2}M_{ik}^{2}$, respectively. Define $R_{ij}=M_{ij}-\sum_{k\neq j}(\beta_{jk}^{T}{\text{X}}_{i})M_{ik}$. By the method of moments, we obtain initial estimates of the graph by minimizing the following objective functions $\sum_{i=n}^{n}(M_{ij}-\sum_{k\neq j}(\beta_{jk}^{T}{\text{X}}_{i})M_{ik}{)}^{2}$ and $\sum_{i=n}^{n}{\left(R_{ij}^{2}-\sigma_{\varepsilon_{j}}^{2}-\sum_{k\neq j}\sigma_{jk}^{2}M_{ik}^{2}\right)}^{2}$ for each *j* with *j* = 1, ⋯, *p*. In order to obtain an initial sparse graph, ℓ_1_-norm penalty can be included to minimize the objective function and obtain initial estimates $\left\{{\tilde{\sigma }}_{jk}^{2}\right\}$, $\left\{{\tilde{\sigma }}_{jk}^{2}\right\}$, and $\left\{{\tilde{\sigma }}_{\varepsilon_{j}}^{2}\right\}$:

(7) $$\begin{array}{l} \sum_{j=1}^{p}\left(\sum_{i=1}^{n}(M_{ij}-\sum_{k\neq j}(\beta_{jk}^{T}{\text{X}}_{i})M_{ik}{)}^{2}+\lambda_{1}\sum_{k\neq j}\|\beta_{jk}{\|}_{1}\right),\\ \sum_{j=1}^{p}\left(\sum_{i=1}^{n}({\tilde{R}}_{ij}^{2}-\sigma_{\varepsilon_{j}}^{2}-\sum_{k\neq j}\sigma_{jk}^{2}M_{ik}^{2}{)}^{2}+\lambda_{2}\sum_{k\neq j}\sigma_{jk}^{2}\right),\;\;\\ \text{subject to}\;\;\sigma_{\varepsilon_{j}}^{2}>0,\;\sigma_{jk}^{2}\geq 0, \end{array}$$

where ${\tilde{R}}_{ij}$ is *R*_*ij*_ with β_*jk*_ replaced by ${\tilde{\beta }}_{jk}$, the parameter estimated from minimizing the first objective function of β at the current iteration. Here we use the same tuning parameter across nodes *j* = 1, ⋯ , *p* for illustration, although in practice node-specific tuning parameter can be used at the price of increasing computational burden. In cases where the topology of the graph varies greatly across nodes, different tuning parameters can be used. Given a regularization path with varying λ_1_ and λ_2_, we select the optimal $\lambda_{1}^{*}$ and $\lambda_{2}^{*}$ using the BIC criteria and apply the corresponding estimates as the initial skeleton. We set the edge (*j, k*) of the initial graph as null if ${\tilde{\beta }}_{jk}=0$ and ${\tilde{\sigma }}_{jk}^{2}=0$.

### 2.2. Algorithms for estimating DAG with mixed-effects model (DAG-MM) and justification

The initial graph, although asymptotically consistent (Meinshausen and Bühlmann, 2006), may not satisfy the DAG constraint due to that estimated ${\hat{\beta }}_{jk}\neq 0$ and ${\hat{\beta }}_{kj}\neq 0$ or ${\hat{\sigma }}_{jk}\neq 0$ and ${\hat{\sigma }}_{kj}\neq 0$. Define graph **A** (set of nodes, edges, and edge strength) as the set of non-null edges $\left\{\left(j,k\right):\sqrt{∥\beta_{jk}{∥}_{2}^{2}/q+\sigma_{jk}^{2}}>0\right\}$ in the skeleton resulting from (7). Let $\theta_{\text{A}}=\beta_{jk},\sigma_{jk}^{2}:\left(j,k\right)\in \text{A};\;\sigma_{\varepsilon_{j}}^{2}:j=1,\cdots \;,p$ be the parameters for graph **A** and *n*_**A**_ be the number of non-zero edges of **A**. To obtain a sparse DAG, a direct approach is to constrain the number of edges in the graph by optimizing a regularized likelihood:

(8) $$\begin{array}{r} \min l_{n}\left(\theta_{\text{A}}\right),\;\text{subject to }\text{A}\text{is a DAG and}n_{\text{A}}<C, \end{array}$$

where *C* is a tuning parameter controlling the number of edges in **A**. The constraints in (8) guarantee the estimated graph is a DAG and also perform edge selection. However, the optimization in (8) is NP-hard, because one needs to evaluate all possible graphs that satisfy the constraint *n*_**A**_<*C*. Furthermore, the computational challenge is elevated due to the acyclic constraint.

Instead, we perform hard-thresholding to approximately solve the ℓ_0_-norm constrained optimization problem in (8). Specifically, after the estimates in ${\hat{\theta }}_{A}$ are obtained for a given graph skeleton **A**, we perform hard-threshold on the estimated edge weights by removing the edge with the smallest $\sqrt{∥{\hat{\beta }}_{jk}{∥}_{2}^{2}/q+{\hat{\sigma }}_{jk}^{2}}$ from **A** and then update the graph **A**. Given an updated graph **A**, we then start from the estimates obtained in the previous iteration and update the estimate ${\hat{\theta }}_{A}$. This procedure continues until some criterion of optimality is met. In our implementation, we use BIC as the criterion to select the optimal graph.

The above procedure can be summarized into a DAG-MM algorithm (described in Algorithm 1). The tasks include identifying graph structure (set of nodes and edges), direction of edges, and edge strength. DAG-MM consists of three main steps: estimation of sparse skeleton and edge strength, edge orientation, and iterative DAG building. In the first step, each node's Markov blanket is identified by penalized likelihood and edge strength is obtained. In the second step, edge orientation is performed by removing directionalities with weak dependence (computed from fixed-effects parameters and variances of random effects). In the third step, an iterative procedure performs edge pruning using the norm of the edge connection strength and searches for the DAG that satisfies the acyclic constraint using a general and fast routine described in a DAG-Checking algorithm (described in Algorithm 2 in Supplementary Material).

Algorithm 1 is computationally efficient for several reasons: the sparse skeleton reduces the search space of DAGs; ranking by the magnitude of edge effects provides search paths in the DAG space; selection criteria BIC is only calculated when the log-likelihood (6) is the correct model (i.e., the acyclic constraint is satisfied); and the optimal graph is selected from candidate DAGs. We observe empirically that the full graph shrinks to a DAG very fast in only a few iterations of the third step. For implementation, we have developed main routines in C++ codes with an R interface (R program available upon request).

**Algorithm 1:** DAG With Mixed Model (DAG-MM)

Sparse skeleton: Estimate an initial sparse graph **A**_*I*_ by solving the objective function (7). Obtain the estimates ${\hat{\theta }}_{A_{I}}$ by minimizing (6) for **A**_*I*_. Edge orientation: Initialize **A**_*R*_ = **A**_*I*_. For (*j, k*) belongs to {(*j, k*):(*j, k*) ∈ **A**_*R*_and(*k, j*) ∈ **A**_*R*_}, prune the initial graph: (a) Calculate $c_{jk}=\sqrt{∥{\hat{\beta }}_{jk}{∥}_{2}^{2}/q+{\hat{\sigma }}_{jk}^{2}}$ and *r*_*jk*_ = *c*_*jk*_/*c*_*kj*_ for all (*j, k*) ∈ {(*j, k*):(*j, k*) ∈ **A**_*R*_and(*k, j*) ∈ **A**_*R*_}. (b) Remove the edge (*j, k*), where (*j, k*) = argmin_*j, k*_*r*_*jk*_; update **A**_*R*_ = **A**_*R*_ \ (*j, k*). (c) Update the estimate ${\hat{\theta }}_{A_{R}}$ by minimizing (6) for **A**_*R*_. Iterative DAG building: Initialize **A**_1_ = **A**_*R*_. For *i* = 1, ⋯, p^*^(*p*−1)/2 or until **A**_*i*_ = ∅, search DAG with hard-thresholding: (a) Update the estimate ${\hat{\theta }}_{{\text{A}}_{i}}$ by minimizing (6) for **A**_*i*_. (b) Calculate BIC if **A**_*i*_ is a DAG. (c) Perform edge pruning by removing the edge (*j, k*) with the smallest $\sqrt{∥{\hat{\beta }}_{jk}{∥}_{2}^{2}/q+{\hat{\sigma }}_{jk}^{2}}$. Obtain the updated graph **A**_*i*+1_ = **A**_*i*_ \ (*j, k*), and check whether **A**_*i*+1_ satisfies acyclic constraint by Algorithm 2 if **A**_*i*_ is not a DAG.

### 2.3. Rationale of DAG-MM algorithm and theoretical result

Essentially DAG-MM uses the likelihood function as the objective function in the optimization and thus belongs to the class of score-based approaches for estimating DAG. Similar to other score-based methods in this class (Heckerman et al., 1995), the search is performed locally at each iteration. The first step provides a sparse skeleton and consistent initial estimators of DAG edge strength through moment estimation, with the magnitude of estimated effects close to the truth parameter values. In the second step, the direction that maximizes the network edge strength is selected. The rationale is that the overall edge strength under the correct direction is greater than the strength under the incorrect one (which is close to null effect). In the third step, the DAG with the lowest BIC objective function is selected. Under the identifiability result in Theorem 1 shown below, the optima is uniquely identified, and the DAG-MM algorithm may converge in a local neighborhood of true parameters.

Next, we prove the identifiability of the DAG-MM procedure. Here we omit the subscript *i* corresponding to subjects. For any matrix *B* = {_β_*jk*_}*j, k*_ and $\Sigma ={\left\{\sigma_{jk}^{2}\right\}}_{j,k}$, we call (*B*, Σ) to be compatible with DAG, denoted by (*B*, Σ)~*DAG*, if the edge pair (*j, k*) such that β_*jk*_≠0 or σ_*jk*_≠0 forms a DAG network. Furthermore, we use *L*(*B*, Σ, θ) to denote the likelihood function associated with (*B*, Σ) using the SEM, where $\theta ={\left(\sigma_{\varepsilon_{1}}^{2},\ldots ,\sigma_{\varepsilon_{p}}^{2}\right)}^{T}$. Note that if (*B*, Σ)~*DAG*, then |*I*−*B*(*X*)−Γ| = 1, so

(9) $$\begin{array}{l} L(B,\Sigma ,\theta )=\exp\{-\sum_{j=1}^{p}[\frac{{\left(M_{j}-\sum_{k\neq j}\left(\beta_{jk}^{T}X\right)M_{k}\right)}^{2}}{\sum_{k\neq j}\sigma_{jk}^{2}M_{k}^{2}+\sigma_{\varepsilon_{j}}^{2}}\\ \;+\log(\sum_{k\neq j}\sigma_{jk}^{2}M_{k}^{2}+\sigma_{\varepsilon_{j}}^{2})]\}. \end{array}$$

In Theorem 1, we prove the identifiability by assuming $\sigma_{\varepsilon_{j}}^{2}>0$ for any *j* = 1, …, *p*.

**Theorem 1**. Assume that *P*(β^*T*^*X* = 0) < 1 for any β≠0, i.e., *X* is full rank with positive probability. Let (*B*_0_, Σ_0_, θ_0_) be the true values in the underlying true DAG, and let *ch*_0_(*k*) denote the set of child nodes of the node *k*. Assume that for all nodes *k*, $\underset{j\in ch_{0}\left(k\right)}{\sum }{\left(\beta_{0jk}^{T}X\right)}^{2}$ is not a constant (heterogeneity assumption) across nodes. Suppose (*B*, Σ, θ) ~ *DAG* and *L*(*B*, Σ, θ) = *L*(*B*_0_, Σ_0_, θ_0_). Then, *B*_0_ = *B*, Σ_0_ = Σ, and $\sigma_{0\varepsilon_{j}}^{2}=\sigma_{\varepsilon_{j}}^{2}$ for *j* = 1, …, *p*.

The proof of the theorem is in the Supplementary Material Section S3. The heterogeneity assumption implies that when there are multiple child nodes, their squared edge strengths from fixed effects are different across parental nodes. When there is a single child node, the edge strengths are different across subpopulations defined by covariates **X**.

## 3. Simulation studies

**Figure 1:**
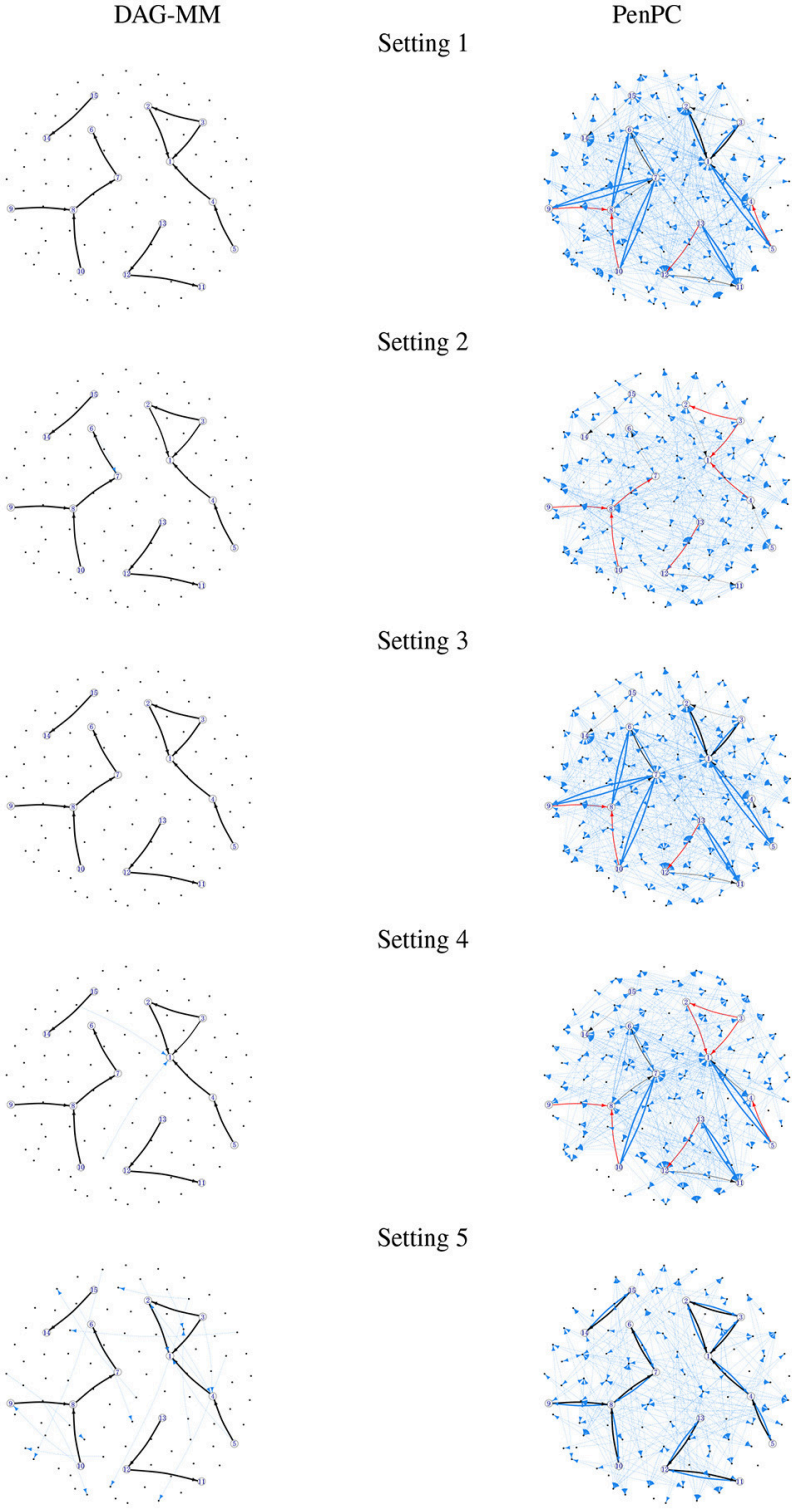
Frequency of edges selected in 100 simulations. Edge width is proportional to the number of times an edge is identified in simulations. Black: true positive edges; Blue: false positive edges; Red: false negative edges (true edges that were never selected).

We performed comprehensive simulations to evaluate DAG-MM with varying sample sizes, *n* = 200, 500, 1, 000, and varying number of nodes, *p* = 20, 50, 100. We let $\sigma_{\varepsilon_{2*j-1}}^{2}=1.0$ and $\sigma_{\varepsilon_{2*j}}^{2}=0.5$, and the dimension of exogenous covariates **X** is 3: two of them are continuous variables that follow the standard normal distribution *N*(0, 1), and the other is a binary variable that follows the Bernoulli distribution, *Bernoulli*(0.5). Note that there are at most *p* * (*p*−1) ^*^ (*q*+1)+*p* parameters to be estimated. For example, the total number of parameters is 1540 when *p* = 100 and *q* = 3. We fixed 12 non-zero edges as shown in Figure 1 (black edges), and the other features were independent noise variables. For the non-null edges, we let β_*jk*_ = (−0.5, 1.0, −1.5) and $\sigma_{jk}^{2}=0.5$. Several settings were considered in our simulations:

1. Fixed effects only: β_*jk*_ = (−0.5, 1.0, −1.5) and $\sigma_{jk}^{2}=0$ for (*j, k*) ∈ **A**^0^.
2. Random effects only: β_*jk*_ = **0** and $\sigma_{jk}^{2}=0.5$ for (*j, k*) ∈ **A**^0^.
3. Mixed effects 1: β_*jk*_ = (−0.5, 1.0, −1.5) and $\sigma_{jk}^{2}=0.5$ for (*j, k*) ∈ **A**^0^.
4. Mixed effects 2: β_*jk*_ = (−0.5, 1.0, −1.5) for (*j, k*) ∈ {(1, 2), (1, 4), (4, 5), (7, 8), (8, 10), (11, 12), (12, 13), (14, 15)} and $\sigma_{jk}^{2}=0.5$ for (*j, k*) ∈ {(1, 2), (1, 3), (1, 4), (2, 3), (6, 7), (8, 9), (8, 10), (12, 13)}.
5. Homogeneous, constant effects without covariates or random effects: we include a column of ones into **X**_*i*_. ${\left(\beta_{jk,2},\ldots ,\beta_{jk,q+1}\right)}^{\prime }=0$, $\sigma_{jk}^{2}=0$, β_*jk*, 1_ = 1 for (*j, k*) ∈ {(1, 2), (1, 4), (4, 5), (7, 8), (8, 10), (12, 13}, and β_*jk*, 1_ = −1 for (*j, k*) ∈ {(1, 3), (2, 3), (6, 7), (8, 9), (11, 12), (14, 15)}.

In each simulation, we compared DAG-MM with the commonly used PC algorithm (Kalisch et al., 2012) and a two-step penalized version of the PC algorithm, penPC (Ha et al., 2016). We used the default settings in R-packages “pcalg" and “penPC" for these alternative methods (e.g., with α = 0.1). The edge selection performance was assessed by the number of true positive (TP) edges and false positive (FP) edges, taking into consideration the direction (i.e., an edge with a wrong direction will be counted as false). To evaluate the estimation of causal effects, we calculated the root sum squared (RSS) error of {${\hat{\beta }}_{jk}$}, {${\hat{\sigma }}_{jk}^{2}$}, and {${\hat{\sigma }}_{\varepsilon_{j}}^{2}$}, which is defined as $RSS\left(\hat{\beta }\right)=\sqrt{\underset{j\neq k}{\sum }∥{\hat{\beta }}_{jk}-\beta_{jk}{∥}_{2}^{2}}$, $RSS\left({\hat{\sigma }}^{2}\right)=\sqrt{\underset{j\neq k}{\sum }{\hat{\sigma }}_{jk}^{2}-\sigma_{jk}^{2}{}^{2}}$, and $RSS\left({\hat{\sigma }}_{\varepsilon }^{2}\right)=\sqrt{\overset{p}{\underset{j=1}{\sum }}{\hat{\sigma }}_{\varepsilon_{j}}^{2}-\sigma_{\varepsilon_{j}}^{2}{}^{2}}$, respectively.

The simulations were repeated 100 times for each setting.

Table 1 summarizes the number of TP and FP edge selections. The initial graph selection (i.e., performing steps 1 and 2 in Algorithm 1) correctly identified the true edges for all settings with TP edges very close to 12, but also selected many FP edges. Starting from the initial graph, the DAG-MM procedure can retain almost all the TP edges and also remove most FP edges, with a FP rate close to 0. Note that there are 9,900 edges in total when *p* = 100, and DAG-MM can still select the 12 true edges from a total of 9,900 edges (0.05%). With a small sample size of *n* = 200, the performance of DAG-MM remains to be satisfactory, except in Setting 2. Setting 2 is more difficult because all edges involve latent effects. DAG-MM selects about 40% of TP edges when *n* = 200 and selects almost all true edges when the sample size increases to *n* = 1, 000, without including FP edges. PC and penPC algorithms are designed for Setting 5 - constant effect without any covariates. As expected, they perform the best for Setting 5 but not other settings, and penPC selects fewer FP edges than PC algorithm due to an initial penalized regression step. However, for Setting 5, DAG-MM significantly outperforms the two PC algorithms in terms of fewer FP. Figure 1 visualizes the number of times (greater than one) that an edge is selected in the simulations. The visualization shows that DAG-MM performs satisfactorily and correctly identifies the true network structure in all settings. In contrast, penPC identifies many edges with incorrect direction and includes many more FP edges.

**Table 1:** Simulation results of graph edge selection performance (TP: average number of true positive edges; FP: average number of false positive edges; FN: average number of false negative edges) using the initial DAG selection, DAG-MM procedure, PC algorithm, and penPC algorithm for various sample sizes *n* and numbers of features *p*.

		**Initial graph**			**DAG-MM**			**PC**			**penPC**		

		*p* = 20	*p* = 50	*p* = 100	*p* = 20	*p* = 50	*p* = 100	*p* = 20	*p* = 50	*p* = 100	*p* = 20	*p* = 50	*p* = 100
**SETTING 1-FIXED EFFECTS ONLY**													
TP	*n* = 200	12.0	12.0	12.0	12.0	12.0	12.0	1.8	1.4	1.2	2.7	2.5	2.4
	*n* = 500	12.0	12.0	12.0	12.0	12.0	12.0	2.0	1.7	1.4	3.0	3.0	3.0
	*n* = 1, 000	12.0	12.0	12.0	12.0	12.0	12.0	2.1	1.6	1.4	3.2	2.9	3.0
FP	*n* = 200	33.1	77.9	162.8	0.2	0.0	0.1	9.3	18.7	48.6	18.1	26.5	46.0
	*n* = 500	32.9	69.7	69.2	0.0	0.0	0.0	9.8	19.9	51.2	19.6	28.1	42.8
	*n* = 1, 000	25.2	44.1	74.1	0.0	0.0	0.0	9.9	20.7	53.2	19.7	28.8	40.7
FN	*n* = 200	0.0	0.0	0.0	0.0	0.0	0.0	10.2	10.6	10.8	9.3	9.5	9.6
	*n* = 500	0.0	0.0	0.0	0.0	0.0	0.0	10.1	10.4	10.6	9.0	9.0	9.0
	*n* = 1, 000	0.0	0.0	0.0	0.0	0.0	0.0	9.9	10.4	10.6	8.8	9.1	9.0
**SETTING 2-RANDOM EFFECTS ONLY**													
TP	*n* = 200	11.5	11.3	10.7	6.9	5.3	3.7	0.6	0.4	0.2	0.6	0.4	0.3
	*n* = 500	12.0	11.9	11.9	10.4	10.3	10.0	0.5	0.3	0.2	0.6	0.3	0.2
	*n* = 1, 000	12.0	12.0	12.0	11.3	11.3	11.3	0.3	0.2	0.1	0.4	0.3	0.2
FP	*n* = 200	57.2	130.5	215.7	1.1	2.2	3.1	3.4	15.4	46.1	4.0	14.5	31.5
	*n* = 500	56.3	96.4	167.0	0.3	0.8	1.4	3.5	15.7	49.7	3.9	13.3	25.9
	*n* = 1, 000	49.8	115.6	109.3	0.0	0.2	0.2	3.6	16.9	51.7	4.3	14.7	25.0
FN	*n* = 200	0.5	0.8	1.3	5.1	6.8	8.3	11.5	11.7	11.8	11.4	11.6	11.7
	*n* = 500	0.0	0.1	0.1	1.6	1.7	2.0	11.5	11.8	11.8	11.4	11.7	11.8
	*n* = 1, 000	0.0	0.0	0.0	0.7	0.7	0.7	11.7	11.8	11.9	11.6	11.7	11.8
**SETTING 3-MIXED EFFECTS 1**													
TP	*n* = 200	12.0	12.0	12.0	12.0	12.0	11.9	1.7	1.4	1.1	2.6	2.5	2.4
	*n* = 500	12.0	12.0	12.0	12.0	12.0	12.0	1.9	1.6	1.4	3.0	2.9	2.8
	*n* = 1, 000	12.0	12.0	12.0	12.0	12.0	12.0	2.1	2.0	1.4	3.1	3.2	3.0
FP	*n* = 200	114.8	228.7	362.5	0.0	0.2	0.7	8.9	18.4	47.5	17.1	26.8	44.1
	*n* = 500	109.6	266.8	431.3	0.0	0.0	0.0	9.2	19.7	51.2	17.9	27.9	41.6
	*n* = 1, 000	138.9	185.8	326.4	0.0	0.0	0.0	9.4	20.2	53.8	18.5	29.1	40.5
FN	*n* = 200	0.0	0.0	0.0	0.0	0.0	0.1	10.3	10.6	10.9	9.4	9.5	9.6
	*n* = 500	0.0	0.0	0.0	0.0	0.0	0.0	10.1	10.5	10.7	9.0	9.2	9.2
	*n* = 1, 000	0.0	0.0	0.0	0.0	0.0	0.0	9.9	10.0	10.6	8.9	8.8	9.0
**SETTING 4-MIXED EFFECTS 2**													
TP	*n* = 200	11.7	11.4	11.2	10.7	10.3	9.8	0.6	0.6	0.4	1.1	1.1	0.9
	*n* = 500	11.9	11.8	11.7	11.6	11.3	11.1	0.5	0.4	0.4	1.0	1.0	0.9
	*n* = 1, 000	12.0	12.0	11.9	11.6	11.6	11.5	0.6	0.5	0.5	1.1	1.2	1.1
FP	*n* = 200	81.7	121.4	237.9	0.6	2.3	5.5	8.0	18.3	47.8	12.2	22.2	41.3
	*n* = 500	56.1	155.5	258.0	0.1	0.4	0.8	8.3	18.9	50.8	12.7	21.6	36.4
	*n* = 1, 000	92.1	96.0	161.6	0.0	0.1	0.1	8.2	19.7	52.4	13.5	22.2	33.8
FN	*n* = 200	0.3	0.6	0.8	1.3	1.7	2.2	11.4	11.4	11.7	10.9	10.9	11.1
	*n* = 500	0.1	0.2	0.3	0.4	0.7	0.9	11.5	11.6	11.6	11.0	11.0	11.1
	*n* = 1, 000	0.0	0.0	0.1	0.4	0.4	0.5	11.4	11.5	11.6	10.9	10.8	10.9
**SETTING 5-HOMOGENEOUS (CONSTANT EFFECT)**													
TP	*n* = 200	12.0	12.0	12.0	11.9	12.0	11.9	11.2	10.8	10.3	11.9	11.9	11.8
	*n* = 500	12.0	12.0	12.0	12.0	12.0	12.0	11.8	11.5	11.0	12.0	12.0	12.0
	*n* = 1, 000	12.0	12.0	12.0	12.0	12.0	12.0	11.9	11.6	11.3	12.0	12.0	12.0
FP	*n* = 200	37.3	24.6	68.9	0.3	1.3	5.0	6.8	14.7	42.6	12.4	19.3	36.9
	*n* = 500	23.2	49.6	22.2	0.2	0.3	0.8	5.7	14.5	43.7	12.6	19.3	33.9
	*n* = 1, 000	36.3	22.6	31.8	0.0	0.7	3.6	5.3	14.5	45.3	12.5	19.2	30.4
FN	*n* = 200	0.0	0.0	0.0	0.1	0.0	0.1	0.8	1.3	1.7	0.1	0.2	0.2
	*n* = 500	0.0	0.0	0.0	0.0	0.0	0.0	0.2	0.5	1.0	0.0	0.0	0.0
	*n* = 1, 000	0.0	0.0	0.0	0.0	0.0	0.0	0.1	0.4	0.8	0.0	0.0	0.0

Next, we examined the estimation performance of the strength of the connection. Table 2 shows the RSS for parameters β, σ^2^, and $\sigma_{\varepsilon }^{2}$. Overall, RSS decreases to small values as sample size *n* increases. The increase in the number of features *p* affects the estimation of variance components σ^2^ and $\sigma_{\varepsilon }^{2}$ more than β. The results may suggest that for large *p*, including more samples improves the estimation performance of the individual-level heterogeneity associated with γ_*ijk*_. The sample size required to obtain stable estimates depends on the true underlying graph (e.g., number of nodes, number of true edges, edge strength and its variability across subjects). In our simulation examples, for a network with 20 nodes and 12 true edges, a small sample size of *n* = 500 gives adequate performance under all five settings. With a larger graph with 100 nodes and 12 true edges, *n* = 1, 000 gives adequate performance.

**Table 2:** Simulation results of root sum-squared (RSS) error of parameters for the connection strength estimated by DAG-MM under various sample sizes *n* and numbers of features *p*.

	β			**σ**^**2**^			$\sigma_{\varepsilon }^{2}$		

	*p* = 20	*p* = 50	*p* = 100	*p* = 20	*p* = 50	*p* = 100	*p* = 20	*p* = 50	*p* = 100
**SETTING 1-FIXED EFFECTS ONLY**									
*n* = 200	0.312	0.305	0.358	0.077	0.087	0.102	0.388	0.576	0.832
*n* = 500	0.184	0.188	0.189	0.045	0.045	0.049	0.232	0.360	0.505
*n* = 1, 000	0.130	0.131	0.130	0.037	0.034	0.032	0.162	0.253	0.357
**SETTING 2-RANDOM EFFECTS ONLY**									
*n* = 200	0.527	0.501	0.479	1.347	1.708	1.977	1.065	1.307	1.601
*n* = 500	0.353	0.369	0.386	0.739	0.815	0.943	0.523	0.631	0.714
*n* = 1, 000	0.254	0.270	0.270	0.461	0.496	0.485	0.294	0.400	0.458
**SETTING 3-MIXED EFFECTS 1**									
*n* = 200	0.606	0.667	1.063	0.635	0.857	4.058	0.523	0.693	0.962
*n* = 500	0.367	0.362	0.363	0.391	0.355	0.348	0.347	0.433	0.564
*n* = 1, 000	0.254	0.261	0.262	0.259	0.264	0.248	0.227	0.300	0.387
**SETTING 4-MIXED EFFECTS 2**									
*n* = 200	0.559	0.624	0.797	0.963	1.336	2.359	0.649	0.888	1.243
*n* = 500	0.333	0.345	0.365	0.478	0.593	0.783	0.375	0.462	0.627
*n* = 1, 000	0.234	0.229	0.233	0.351	0.389	0.427	0.252	0.333	0.436
**SETTING 5-HOMOGENEOUS (CONSTANT EFFECT)**									
*n* = 200	0.358	0.348	0.447	0.125	0.303	0.837	0.479	0.647	0.936
*n* = 500	0.157	0.166	0.157	0.073	0.112	0.205	0.251	0.389	0.530
*n* = 1, 000	0.098	0.143	0.226	0.048	0.054	0.093	0.172	0.269	0.363

The computing time for DAG-MM is highly manageable. For example, in simulation Setting 5, the running time (averaged over 100 replicates) for simulated data with *n* = 1, 000 is 0.4 s for *p* = 20, 1.2 s for *p* = 50, and 4.4 s for *p* = 100, compared to 3.2, 16.8, and 66.5 s, respectively, for the penPC algorithm.

## 4. Applications to protein signaling network and brain dependence network

### 4.1. Protein signaling network

Our first application involved a study that examined the interaction between major mitogen-activated protein kinase (MAPK) pathways in human CD4+ T cells. Using intracellular multicolor flow cytometry, single-cell protein expression levels were measured for 11 proteins in the MAPK pathways in Sachs et al. (2005). Six experiments were performed using different stimuli, each targeting a different protein in the selected pathway (Sachs et al., 2005), and thus both interventional and unperturbed observational data were available for our application. Various data-driven methods were proposed to estimate the protein signaling networks, including Bayesian network analyses (Sachs et al., 2005; Mooij and Heskes, 2013) and ICP using combined interventional and observational data (Meinshausen et al., 2016), and results were compared with a consensus network in the literature (Mooij and Heskes, 2013; Meinshausen et al., 2016). The datasets are available from https://github.com/lawrennd/rca/tree/master/matlab/PROTSIG_DATA. There were a total of 7392 available measurements in both perturbed and unperturbed settings in the Experiments 1-9, which were used by Sachs et al. (2005) and Meinshausen et al. (2016) in their main analyses.

In our analyses, we applied DAG-MM to learn the causal signaling network using unperturbed, observational data only, including anti-CD3 + anti-CD28, anti-CD3/CD28 + ICAM-2, and anti-CD3/CD28 + LY294002. These experiments did not directly intervene on the activity of 11 proteins. The observational data consisted of 2,594 observations and were pre-processed using a standard arcsinh transformation for biological interpretability. DAG-MM with fixed effects only (DAG-MM1) and with mixed effects (DAG-MM2) were applied. Meinshausen et al. (2016) used a subset of 8 of the 9 environments and one of the environment is considered as observational data without external interventions. Each environment contains between 700 and 1,000 samples. There are 11 nodes in the signaling network. Sachs et al. (2005) identified 17 edges and Meinshausen et al. (2016) identified 7 edges by ICP and 13 edges by hiddenICP. All these identified edges were summarized in the Supplementary Table S1 in Meinshausen et al. (2016). Our results were compared with those obtained using the PC algorithm as reported in Kalisch et al. (2012) and with ICP as reported in Meinshausen et al. (2016) for both interventional and observational data.

**Figure 2:**
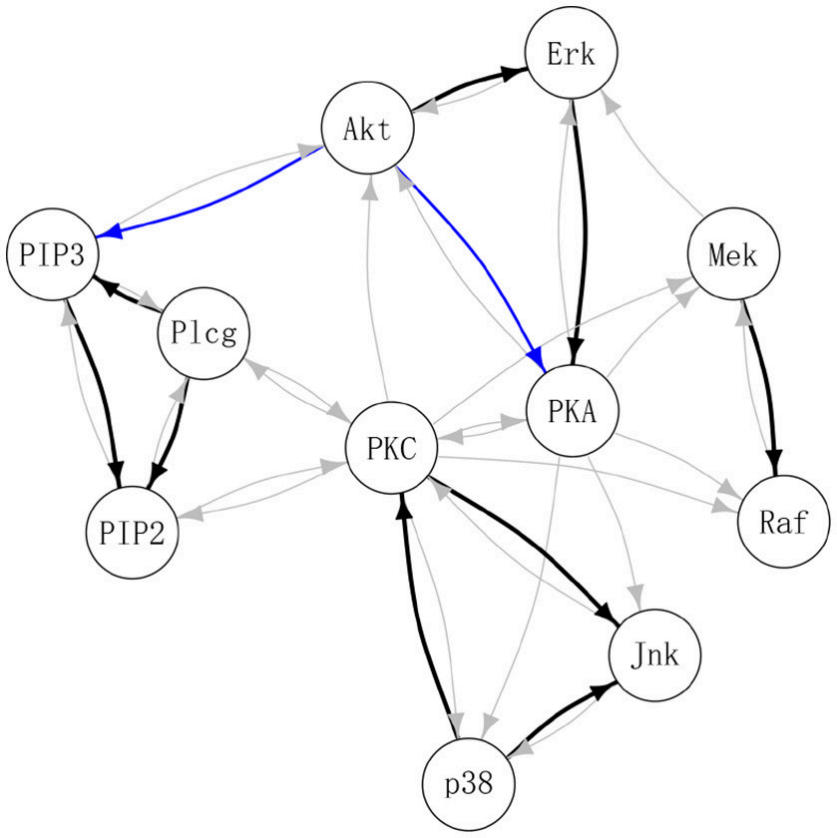
Estimated protein signaling network. Black: edges identified by DAG-MM2 and also reported previously (see summary Table S1 in Meinshausen et al., 2016); Blue: edges are identified by DAG-MM2 but not reported previously; Gray: edges previously reported edges but not identified by DAG-MM2.

Table 3 summarizes the number of selected edges by each method and whether these edges were also previously reported in the literature. Treating the edges previously identified and reported in Supplementary Table S1 of Meinshausen et al. (2016) as “gold standard,” DAG-MM2 reduces the number of FP edges to a greater extent than DAG-MM1. PC and ICP identified a similar number of true positive edges as DAG-MM2, but with a higher number of FP edges. In Figure 2, we show edges identified previously in Meinshausen et al. (2016) or by our methods (edges identified elsewhere, e.g., between AKT and RAF are not shown). We focus on comparing DAG-MM2 with ICP. The skeleton of DAG-MM2 and ICP is almost identical, with DAG-MM2 identifying one more edge, Plcg → PIP3. Two edges were in the reverse direction of those reported in literature, which might due to feedback loops that are expected to be present in this system (Meinshausen et al., 2016). The striking similarity of DAG-MM2 identified from observational data alone and ICP using interventional data suggests robustness and the ability of the former to infer causal relationships from observational data by including random effects.

**Table 3:** Comparison with previously identified causal relationships.

**Reported^†^**	**PC**	**ICP**	**DAG-MM1**	**DAG-MM2**
Yes	8	10	8	9
No	4	5	10	2^*^

*Total number of edges previously identified in the literature is 34 (see summary Table S1 in Meinshausen et al., 2016). ICP (Meinshausen et al., 2016) used both observational and interventional data. Proposed DAG-MM1 (fixed effects only) and DAG-MM2 (mixed effects) used only observational data*.

† *Whether an edge was previously reported in the literature*.

* *Edges in reverse direction of those reported in the literature*.

### 4.2. Brain gray matter atrophy dependence network

Our second application involved a study on atrophy networks in the brains of patients with HD. HD is a monogenic neurodegenerative disorder caused by an expansion of the CAG trinucleotide (≥36) in the *huntingtin* gene (O'Donovan, 1993). The hallmark of HD neuropathology is brain atrophy, in terms of gray matter loss within the striatum and white matter loss around the striatum (Ross et al., 2014). While evidence shows that selective brain regions undergo atrophy at different rates (Paulsen et al., 2014), it is unknown how these regional atrophies depend on one another and act together as disease progresses. In this application, we aimed to construct brain atrophy dependence networks using data collected from a large natural-history study of HD progression, PREDICT-HD (Paulsen et al., 2014), and we aimed to corroborate findings in an independent study, TRACK-ON (Klöppel et al., 2015). Subcortical gray matter loss of volume and gray matter cortical thinning were considered as measures of brain atrophy and hallmarks of HD. Thus, we examined dependencies between rates of volume loss and cortical thinning in different brain regions.

For the PREDICT-HD study, we included individuals who carried an expansion of the CAG trinucleotide in the hungtington gene and thus were at risk of HD but had not been diagnosed at baseline. Data consisted of 824 subjects with 68 cortical regions of interest (ROI) and 22 subcortical ROIs measured by structural magnetic resonance imaging (MRI). Longitudinal assessments were obtained from these subjects with a median follow-up period of 3.9 years. The details of MRI data segmentation, preprocessing, and study design are in Paulsen et al. (2014). A linear mixed-effects model with subject-specific random intercepts and random slopes was used to estimate the rate of volumetric change and the rate of cortical thickness change at each ROI for each subject. Rates of change at ROIs form the nodes in the brain atrophy dependence network. Because CAG repeats and age are two variables with substantial contribution to HD, a covariate based on the CAG-age product (CAPs score in Zhang et al., 2011) was created to indicate a subject's risk of receiving a diagnosis of HD (low, medium, and high risk). Baseline age was dichotomized into two groups (young vs. old) based on the median split. A total of seven covariates was included (high risk, medium risk, baseline age group, sex, and baseline clinical measures: total functioning capacity [TFC], total motor score [TMS], symbol digit modalities test [SDMT]).

Potentially there are 462 edges (involving 4,180 parameters) for the subcortical gray matter volumetric atrophy network and 4,556 potential edges (involving 41,072 parameters) for the cortical gray matter thickness network. The proposed DAG-MM identified 5 connections (Supplementary Material Section S4, Table S1) from the subcortical network (e.g., left thalamus to right accumbens, and right pallidum to left putamen), which suggests that most subcortical ROI atrophy rates do not depend on other ROIs. In contrast, a denser network was identified for the cortical thickness network, with 58 connections identified (Supplementary Material Section S4, Table S2), suggesting that cortical thinning acts in a more concerted fashion, consistent with the neuroimaging literature on cortical networks in HD (He et al., 2008). PenPC identified a very dense network for both subcortical volumes (92 edges) and cortical thickness networks (480 edges). Due to its non-sparseness and difficulty in interpretation, we omit results from PenPC and report DAG-MM in the subsequent presentation.

**Figure 3:**
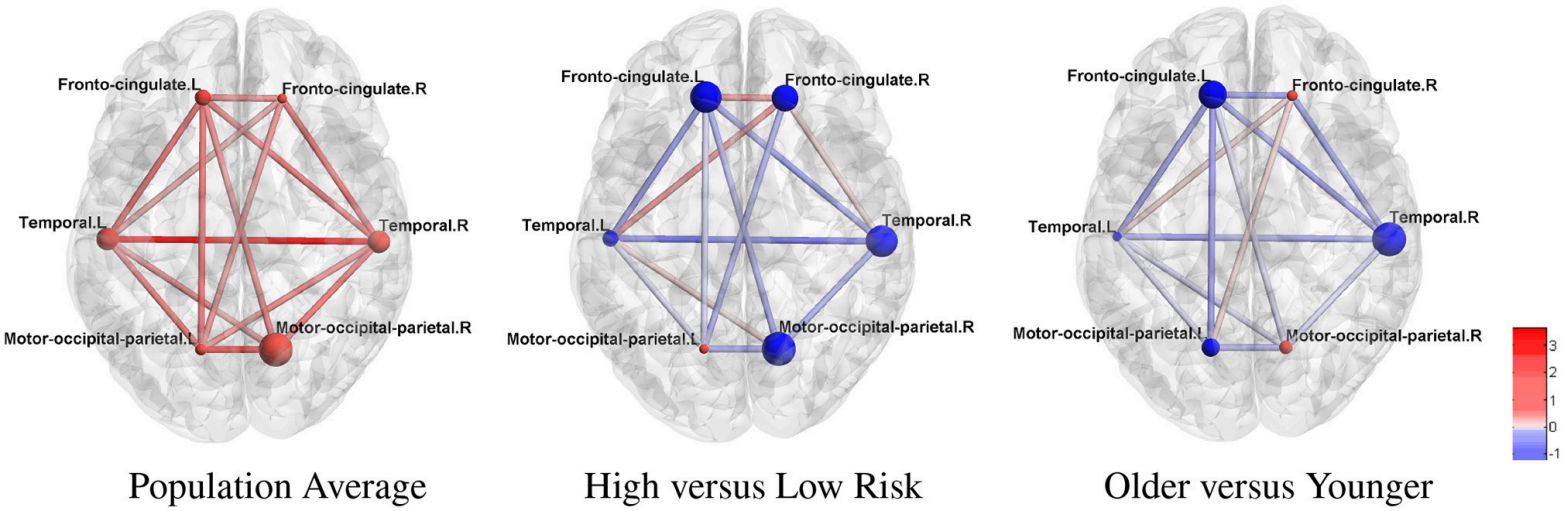
Estimated cortical thickness atrophy dependence network (organized into modules). The node size is proportional to the intra-modular connection strength (edge effects) and scaled within each subfigure. Red nodes: positive effects. Blue nodes: negative effects.

ROIs were further organized into modules related to HD pathology as in McColgan et al. (2017) for better interpretation. We present these results in Figure 3, where the modular-wise strength of the connection was computed as the total strength of connections within a module [summation of β_*jk*_ between all pairs of connected nodes (*j, k*) in the same module] or between two modules (summation of β_*jk*_ between all pairs of connected nodes (*j, k*) for *j* in one module and *k* in the other). Figure 3 shows that the two strongest connections in the average modular graph (with covariates fixed at the sample averages) are the inter-hemispheric links between the left and right temporal regions and between the left and right motor-occipital-parietal regions. For within-modular connection, the right side motor-occipital-parietal module has the strongest strength.

Using the estimated parameters (e.g., **β**_*jk*_) from model (2) and (3), we also examined differences between the networks for high-risk group vs. low-risk group, and medium-risk vs. low-risk (other covariates fixed at the sample average). The differential edge strength of the graphs for subjects in two groups was computed based on $\beta_{jk}^{T}X_{1}-\beta_{jk}^{T}X_{2}$, where ***X*_1_** and ***X*_2_** were covariates for subjects in each group. For the high- vs. low-risk group comparison (Figure 3), the largest difference is in the inter-hemispheric temporal regions. Most within-module and between-module connections show a loss of strength in the high-risk group. For example, a large loss of intra-modular connections within the right motor-occipital-parietal, right temporal, left fronto-cingulate is seen. A loss of between-module connections is observed between the left and right motor-occipital-parietal regions and between the left fronto-cingulate and left and right temporal regions. A minor gain of connection is seen within and between a few modules. A similar trend with a milder effect is present for most connections when contrasting medium-risk and low-risk groups. When comparing older adults with younger adults, most connections show a loss of strength in the older group (Figure 3). The largest loss in the intra-modular connections is in the right temporal region. A loss of between-module connections occurs between the left and right fronto-cingulate regions, between the left fronto-cingulate and left and right temporal regions, between the left fronto-cingulate and left motor-occipital-parietal regions, and between the right fronto-cingulate and right temporal regions.

In Supplementary Material Section S4, Figure S1, we show the node-wise DAGs and the difference of the estimated network between groups with different baseline risk of HD diagnosis. At the nodal level, we see a loss of connection in the high-risk group and older group in a large number of links. The connection with the largest difference is L.caudalmiddlefrontal ⇒ L.rostralmiddlefrontal (based on L2-norm). When effects are aggregated from nodes within modules, group differences are more apparent (Figure 3). The strength of connections between nodes is summarized in Supplementary Material Section S4, Tables S1, S2. Among all covariates, the three covariates with the largest effects aggregated across all connections (based on *L*_2_-norm) are high-low risk group contrast, medium-low risk contrast, and older-younger adult contrast. Substantial heterogeneity of connections due to latent factors not captured by covariates is observed for almost all links (represented by σ^2^ in Supplementary Material Section S4, Tables S1, S2). We show the variation of the heterogeneous effects (standard deviation: σ_*jk*_) of connections in Supplementary Material Section S4, Figure S1. The connection with the highest variation is L.caudalmiddlefrontal ⇒ L.posteriorcingulate. This analysis demonstrates substantial heterogeneity of the brain dependence networks among individuals.

#### 4.2.1. A validation study using independent samples

We sought to corroborate our estimated cortical gray matter network using white matter cortical connectivity network data obtained from an independent study, TRACK-ON (Klöppel et al., 2015; McColgan et al., 2017). TRACK-ON is a longitudinal study of premanifest HD, with 84 subjects and a median follow-up length of 1.89 years. White matter structural connection network was constructed from diffusion tensor imaging (DTI) technology, and connection strengths between pairs of nodes were computed by probabilistic tractography. A similar algorithm as PREDICT-HD was used to define regions of interest, and the same method that was used to partition nodes into HD pathology also informed modules (McColgan et al., 2017). Detailed information on the study design and data pre-processing can be found in McColgan et al. (2017). With longitudinal DTI measurements available, a linear mixed-effects model was used to compute the rate of change in connections between nodes and their *p*-values. Baseline connection, CAG, age, gender, motor score, SDMT, and TFC were included as covariates. Nodes were classified into modules by the same method as the structural MRI network. Inter-modular connection was defined as present if at least *c* pairs (*c* = 1, 2) of nodes (each node resides in the module being considered) were connected after the false discovery rate (FDR) correction (*q* < 0.1). Presence of intra-modular connection was defined similarly based on the number of pairs of nodes connected (with *q* < 0.1) within a module. In total, 30 white matter atrophy connections were identified after the FDR correction.

Supplementary Material Section S4, Table S3 summarizes the module-wise white matter structural connectivity network estimated from the DTI technology. The average modular gray matter atrophy network and the white matter connection network both indicate a strong intra-modular connection in the right-side motor-occipital-parietal region and a strong interhemispheric connection in the left and right motor-occipital-parietal regions, whereas a weak connection (or no connection for the white matter network) was present in the left side of the same module. For some of the other four modules, the intra-modular connection strength for gray matter and white matter appears to be complementary: a stronger link in the former corresponds to a weaker link in the latter. For example, connections between the right temporal and right motor-occipital-parietal regions and between the left and right temporal regions show a moderate to strong dependence in the gray matter network, but are absent in the white matter network. The link between the right-fronto-cingulate region is strong in the white matter network, but weak in the gray matter network. These observations might suggest a mechanism that constrains the total modular connections in the gray matter and white matter networks; thus, a strong connection in one correlates with a weak connection in the other.

We evaluated the consistency of the gray matter cortical network (obtained by DAG-MM2 statistical modeling) with the white matter cortical structural connectivity network (directly measured by DTI technology). The overall operational characteristics of the gray matter network are reported in Table 4, treating the white matter network as the reference since white matter connections were directly measured by DTI. Due to a potential complementary effect on the total number of connections between and within modules, the number of connections in the gray matter and white matter networks is negatively correlated. Thus, we computed the sensitivity as *P*(*L* ≤ *l*|*C*≥*c*), where *L* denotes the number of links in the gray matter network, and *C* denotes the number of links in the white matter network. We fixed the connectivity threshold of the white matter network at *c* = 1 or *c* = 2, and we evaluated the overall consistency of the gray matter network across all levels of threshold *l* by computing the AUC across *l*. The AUC is 0.80 (95%CI: 0.61, 0.99) at *c* = 1 and 0.75 (95%CI: 0.48, 1.00) at *c* = 2. Using a higher threshold *c* increases sensitivity, but with a slightly decreased specificity and a slightly lower AUC. These results show that at the modular level, the gray matter cortical atrophy network estimated by DAG-MM has adequate consistency with the white matter structural connectivity network.

**Table 4:** Operating characteristics of cortical gray matter atrophy dependence network evaluated against the white matter structural connectivity network treated as the reference.

**c**	**AUC (95%CI)**	**Sensitivity**	**Specificity**	**PPV**
1	0.80 (0.61, 0.99)	0.57	1.00	1.00
2	0.75 (0.48, 1.00)	0.75	0.85	0.75

McColgan et al. (2017) reported a modular white matter network obtained by comparing connectivity in patients with HD and healthy controls and applying FDR adjustment (Figure 2 in McColgan et al., 2017). When we compare our results to theirs, we see similarity, in terms of connections between the left and right temporal regions and between the left and right motor-occipital-parietal regions.

## 5. Discussion

In this article, we propose a statistical framework for estimating DAGs with mixed effects in multi-dimensional settings, referred to as DAG-MM. The framework captures covariate-dependent causal effects, along with residual effect modification, by building a series of mSEMs. Our framework is a two-stage approach, which first obtains a sparse initial skeleton (undirected graph) and then searches for DAG through a solution path within the selected skeleton and an easily implemented DAG checking procedure. The DAG-MM method is computationally efficient and has shown satisfactory performance, especially for edge selection and orientation, in both simulation studies and real-data applications. The advantage arises when taking into account the covariate-dependent structure and residual heterogeneous effects through the use of random variables. Specifically, the joint distribution of the nodes in model (2) are non-Gaussian due to these random effects and their multiplicative form with the other nodes. This asymmetry in the joint distribution permits the identification of causal relationships from observational data, which we formally prove in Theorem 1. We note that the edge orientation is more accurate than PC and its derivatives, which assume a symmetric joint distribution. For computation, the regularized likelihood-based approach identifies a sparse skeleton in an efficient fashion.

In the analyses of brain atrophy dependence network in patients with HD, some modules of the gray matter network estimated from the PREDICT-HD study share similarity with the white matter connectivity network estimated from an independent study. For some other modules, the results suggest a complementary mechanism that constrains the total modular-wise connections in gray and white matter networks. In the second application, the protein signaling network constructed from DAG-MM with observational data and invariance causal prediction (ICP) with interventional data (Meinshausen et al., 2016) is highly similar. The latter approach assumes causal relationships remain invariant under interventions that do not directly target a cause. This similarity suggests that the random effects in mSEM may serve a similar role as a random perturbation of the node distribution. Under the invariance assumption, the true causal effects are stable under such perturbation, and thus, DAG-MM generates similar results as ICP, but with only observational data.

The network structure among nodes can be further parameterized to incorporate prior information about the causal effects. For example, the knowledge on pathways in the gene regulatory network available in public databases or discoverable in published literature can be included by removing or adding the edge between nodes *j* and *k* or by restricting the edge direction from *j* to *k*. Model (3) can handle this structure by specifying some values of β_*jk*_ or/and $\sigma_{jk}^{2}$ as zero. In addition, exploring other methods of edge orientation including Bayesian model choice methods is worth future research. Another extension is to analyze temporal data **M**_*i*_ with two time points *t*_0_ and *t*_1_, where the desirable temporal ordering corresponds to removing all edges from **M**_*i*_(*t*_1_) to **M**_*i*_(*t*_0_) and modeling the effect from **M**_*i*_(*t*_0_) to **M**_*i*_(*t*_1_).

DAG-MM can be extended to handle multiple types of data, including neuroimaging, protein, and other biomarker measures of different scales, in a regression framework by choosing the appropriate regression for each data type. When the dimension of covariates **X** is high (e.g., large number of genomic measures), feature selection can be imposed on β in order to choose important covariates. Here, we use mSEMs to estimate network connectivity, but we did not differentiate the fixed effects from the random effects. Our main algorithm is a backward selection method and does not allow edge addition. To overcome this issue, one may start DAG-MM from multiple skeletons, which is an approach that provides a more stable edge selection. Other interesting extensions include direct modeling of a dynamic network among **M**(*t*) to allow for time-varying network structure and associate network connections with clinical outcomes. Lastly, the inference of a subject-specific graph (e.g., *p*-values and confidence intervals) can be based on bootstrap (i.e., bootstrap data *B* times, and construct bootstrap confidence interval for DAG edge strength). However, a formal treatment of inference procedure is a topic worth future research.

## Author contributions

YW and DZ designed and oversaw the study. XL and SX developed algorithm, implemented the study, and carried out the statistical analysis. PM, ST, and RS provided DTI data, discussed results, and gave the biological insights. All authors participated in writing the manuscript.

### Conflict of interest statement

The authors declare that the research was conducted in the absence of any commercial or financial relationships that could be construed as a potential conflict of interest.

## References

[B1] Aragam B. Zhou Q. (2015). Concave penalized estimation of sparse Gaussian Bayesian networks. J. Mach. Learn. Res. 16, 2273–2328.

[B2] Bohlken M. M. Brouwer R. M. Mandl R. C. Van den Heuvel M. P. Hedman A. M. De Hert M.. (2016). Structural brain connectivity as a genetic marker for Schizophrenia. JAMA Psychiatry 73, 11–19. 10.1001/jamapsychiatry.2015.1925 26606729

[B3] Brown J. A. Terashima K. H. Burggren A. C. Ercoli L. M. Miller K. J. Small G. W.. (2011). Brain network local interconnectivity loss in aging APOE-4 allele carriers. Proc. Natl. Acad. Sci. U.S.A. 108, 20760–20765. 10.1073/pnas.1109038108 22106308 PMC3251140

[B4] Cai T. T. Li H. Liu W. Xie J. (2013). Covariate-adjusted precision matrix estimation with an application in genetical genomics. Biometrika 100, 139–156. 10.1093/biomet/ass058 28316337 PMC5351557

[B5] Cheng J. Levina E. Wang P. Zhu J. (2014). A sparse Ising model with covariates. Biometrics 70, 943–953. 10.1111/biom.12202 25099186 PMC4425428

[B6] Colombo D. Maathuis M. H. (2014). Order-independent constraint-based causal structure learning. J. Mach. Learn. Res. 15, 3741–3782.

[B7] Fleeson W. Furr R. M. Arnold E. M. (2010). An agenda for symptom-based research. Behav. Brain Sci. 33, 157–157. 10.1017/S0140525X10000750 20584375

[B8] Friston K. J. (2011). Functional and effective connectivity: a review. Brain Connect. 1, 13–36. 10.1089/brain.2011.0008 22432952

[B9] Guo J. Cheng J. Levina E. Michailidis G. Zhu J. (2015). Estimating heterogeneous graphical models for discrete data with an application to roll call voting. Ann. Appl. Stat. 9:821. 10.1214/13-AOAS700 27182289 PMC4865269

[B10] Ha M. J. Sun W. Xie J. (2016). Penpc: a two-step approach to estimate the skeletons of high-dimensional directed acyclic graphs. Biometrics 72, 146–155. 10.1111/biom.12415 26406114 PMC4808501

[B11] Han S. W. Chen G. Cheon M.-S. Zhong H. (2016). Estimation of directed acyclic graphs through two-stage adaptive Lasso for gene network inference. J. Am. Stat. Assoc. 111, 1004–1019. 10.1080/01621459.2016.1142880 28239216 PMC5322863

[B12] He Y. Chen Z. Evans A. (2008). Structural insights into aberrant topological patterns of large-scale cortical networks in Alzheimer's disease. J. Neurosci. 28, 4756–4766. 10.1523/JNEUROSCI.0141-08.2008 18448652 PMC6670444

[B13] Heckerman D. Geiger D. Chickering D. M. (1995). Learning bayesian networks: the combination of knowledge and statistical data. Mach. Learn. 20, 197–243. 10.1007/BF00994016

[B14] Kalisch M. Bühlmann P. (2007). Estimating high-dimensional directed acyclic graphs with the pc-algorithm. J. Mach. Learn. Res. 8, 613–636.

[B15] Kalisch M. Mächler M. Colombo D. Maathuis M. H. Bühlmann P. (2012). Causal inference using graphical models with the R package pcalg. J. Stat. Softw. 47, 1–26. 10.18637/jss.v047.i11

[B16] Klöppel S. Gregory S. Scheller E. Minkova L. Razi A. Durr A.. (2015). Compensation in preclinical Huntington's disease: evidence from the TRACK-ON HD study. EBioMedicine 2, 1420–1429. 10.1016/j.ebiom.2015.08.002 26629536 PMC4634199

[B17] Langfelder P. Cantle J. P. Chatzopoulou D. Wang N. Gao F. Al-Ramahi I.. (2016). Integrated genomics and proteomics define huntingtin CAG length-dependent networks in mice. Nat. Neurosci. 19, 623–633. 10.1038/nn.4256 26900923 PMC5984042

[B18] Luo R. Zhao H. (2011). Bayesian hierarchical modeling for signaling pathway inference from single cell interventional data. Ann. Appl. Stat. 5:725. 10.1214/10-AOAS425 22162986 PMC3233205

[B19] McColgan P. Seunarine K. K. Gregory S. Razi A. Papoutsi M. Long J. D.. (2017). Topological length of white matter connections predicts their rate of atrophy in premanifest Huntington's disease. JCI Insight 2:e92641. 10.1172/jci.insight.92641 28422761 PMC5396531

[B20] Meinshausen N. Bühlmann P. (2006). High-dimensional graphs and variable selection with the lasso. Ann. Stat. 34, 1436–1462. 10.1214/009053606000000281

[B21] Meinshausen N. Hauser A. Mooij J. M. Peters J. Versteeg P. Bühlmann P. (2016). Methods for causal inference from gene perturbation experiments and validation. Proc. Natl. Acad. Sci. U.S.A. 113, 7361–7368. 10.1073/pnas.1510493113 27382150 PMC4941490

[B22] Mooij J. Heskes T. (2013). Cyclic causal discovery from continuous equilibrium data. arXiv:1309.6849 *[Preprint]*.

[B23] O'Donovan M. C. (1993). A novel gene containing a trinucleotide repeat that is expanded and unstable on Huntington's disease chromosomes. Cell 72, 971–983. 10.1016/0092-8674(93)90585-E 8458085

[B24] Paulsen J. S. Long J. D. Johnson H. J. Aylward E. H. Ross C. A. Williams J. K.. (2014). Clinical and biomarker changes in premanifest Huntington disease show trial feasibility: a decade of the PREDICT-HD study. Front. Aging Neurosci. 6:78. 10.3389/fnagi.2014.00078 24795630 PMC4000999

[B25] Pearl J. (2009). Causality: Models, Reasoning, and Inference, 2nd Edn. New York, NY: Cambridge University Press.

[B26] Robinson R. W. (1971). Counting labeled acyclic digraphs, in New Directions in the Theory of Graphs: Proc. Third Ann Arbor Conference on Graph Theory, ed Harary F. (New York, NY: Academic Press), 239–273.

[B27] Ross C. A. Aylward E. H. Wild E. J. Langbehn D. R. Long J. D. Warner J. H.. (2014). Huntington disease: natural history, biomarkers and prospects for therapeutics. Nat. Rev. Neurol. 10, 204–216. 10.1038/nrneurol.2014.24 24614516

[B28] Sachs K. Perez O. Pe'er D. Lauffenburger D. A. Nolan G. P. (2005). Causal protein-signaling networks derived from multiparameter single-cell data. Science 308, 523–529. 10.1126/science.1105809 15845847

[B29] Shimizu S. Bollen K. (2014). Bayesian estimation of causal direction in acyclic structural equation models with individual-specific confounder variables and non-gaussian distributions. J. Mach. Learn. Res. 15, 2629–2652. PMC6688762 31402848

[B30] Shimizu S. Hoyer P. O. Hyvärinen A. Kerminen A. (2006). A linear non-Gaussian acyclic model for causal discovery. J. Mach. Learn. Res. 7, 2003–2030.

[B31] Shojaie A. Michailidis G. (2010). Penalized likelihood methods for estimation of sparse high-dimensional directed acyclic graphs. Biometrika 97, 519–538. 10.1093/biomet/asq038 22434937 PMC3254233

[B32] Smith S. M. Miller K. L. Salimi-Khorshidi G. Webster M. Beckmann C. F. Nichols T. E.. (2011). Network modelling methods for fMRI. Neuroimage 54, 875–891. 10.1016/j.neuroimage.2010.08.063 20817103

[B33] Spirtes P. Glymour C. N. Scheines R. (2000). Causation, Prediction, and Search. Cambridge, MA: MIT Press.

[B34] Ud-Dean S. M. Heise S. Klamt S. Gunawan R. (2016). Trace+: ensemble inference of gene regulatory networks from transcriptional expression profiles of gene knock-out experiments. BMC Bioinformatics 17:252. 10.1186/s12859-016-1137-z 27342648 PMC4919846

[B35] Woodward J. (2005). Making Things Happen: A Theory of Causal Explanation. New York, NY: Oxford University Press.

[B36] Yin J. Li H. (2011). A sparse conditional Gaussian graphical model for analysis of genetical genomics data. Ann. Appl. Stat. 5:2630. 22905077 10.1214/11-AOAS494 PMC3419502

[B37] Yuan Y. Shen X. Pan W. (2012). Maximum likelihood estimation over directed acyclic Gaussian graphs. Stat. Anal. Data Mining 5, 523–530. 10.1002/sam.11168 24358074 PMC3866136

[B38] Zhang Y. Long J. D. Mills J. A. Warner J. H. Lu W. Paulsen J. S. (2011). Indexing disease progression at study entry with individuals at-risk for Huntington disease. Am. J. Med. Genet. B 156, 751–763. 10.1002/ajmg.b.31232 PMC3174494 21858921

