# Supplementary material for: Learning Subject-Specific Directed Acyclic Graphs With Mixed Effects Structural Equation Models From Observational Data
Source: Front Genet. 2018 Oct 2;9:430. doi: 10.3389/fgene.2018.00430 (PMC6176748; doi:10.3389/fgene.2018.00430)

# *Supplementary Material*:
# Learning Subject-Specific with Mixed Effects Structural Equation Models from Observational Data

## 1 S1: DETAILED DERIVATIONS OF THE LIKELIHOOD FUNCTION

Given the random effects $\Gamma_i$, move $\big(\boldsymbol{B}(\boldsymbol{X}_i)+\Gamma_i\big)\boldsymbol{M}_i$ in formula (3) to the left side, we can obtain that

$$\boldsymbol{M}_i = \big(\boldsymbol{I}-\boldsymbol{B}(\boldsymbol{X}_i)-\Gamma_i\big)^{-1}\boldsymbol{\varepsilon}_i$$

$\boldsymbol{\varepsilon}_i$ follows $N(\boldsymbol{0},\boldsymbol{E})$, where $\mathrm{Cov}[\boldsymbol{\varepsilon}_i]=\boldsymbol{E}$ is a diagonal matrix of $\sigma^2_{\varepsilon_j}$.
Therefore, $\boldsymbol{M}_i$ given $\Gamma_i$ follows $N(\boldsymbol{0},(\boldsymbol{I}-\boldsymbol{B}(\boldsymbol{X}_i)-\Gamma_i)^{-1}\boldsymbol{E}(\boldsymbol{I}-\boldsymbol{B}(\boldsymbol{X}_i)-\Gamma_i)^{-T})$, and

$$\begin{aligned}
&p(\boldsymbol{M}_i;\boldsymbol{X}_i|\Gamma_i) \propto |(\boldsymbol{I}-\boldsymbol{B}(\boldsymbol{X}_i)-\Gamma_i)^{-1}\boldsymbol{E}(\boldsymbol{I}-\boldsymbol{B}(\boldsymbol{X}_i)-\Gamma_i)^{-T}|^{-1/2}\times\\
&\exp\left(-\frac{1}{2}\boldsymbol{M}_i^T(\boldsymbol{I}-\boldsymbol{B}(\boldsymbol{X}_i)-\Gamma_i)^T\boldsymbol{E}^{-1}(\boldsymbol{I}-\boldsymbol{B}(\boldsymbol{X}_i)-\Gamma_i)\boldsymbol{M}_i\right)\\
&\propto |\boldsymbol{E}|^{-1/2}|\boldsymbol{I}-\boldsymbol{B}(\boldsymbol{X}_i)-\Gamma_i|\times\exp\left(-\frac{1}{2}\boldsymbol{M}_i^T(\boldsymbol{I}-\boldsymbol{B}(\boldsymbol{X}_i)-\Gamma_i)^T\boldsymbol{E}^{-1}(\boldsymbol{I}-\boldsymbol{B}(\boldsymbol{X}_i)-\Gamma_i)\boldsymbol{M}_i\right).
\end{aligned}$$

To show how to obtain the log-likelihood function (6), let $\boldsymbol{R}_i=(I-\boldsymbol{B}(\boldsymbol{X}_i))\boldsymbol{M}_i$, and note

$$\begin{aligned}
&\boldsymbol{M}_i^T(I-\boldsymbol{B}(\boldsymbol{X}_i)-\Gamma_i)^T\boldsymbol{E}^{-1}(I-\boldsymbol{B}(\boldsymbol{X}_i)-\Gamma_i)\boldsymbol{M}_i\\
&=(\boldsymbol{R}_i-\Gamma_i\boldsymbol{M}_i)^T\boldsymbol{E}^{-1}(\boldsymbol{R}_i-\Gamma_i\boldsymbol{M}_i)\\
&=\boldsymbol{R}_i^T\boldsymbol{E}^{-1}\boldsymbol{R}_i-2\boldsymbol{R}_i^T\boldsymbol{E}^{-1}\Gamma_i\boldsymbol{M}_i+\boldsymbol{M}_i^T\Gamma_i^T\boldsymbol{E}^{-1}\Gamma_i\boldsymbol{M}_i\\
&=\boldsymbol{R}_i^T\boldsymbol{E}^{-1}\boldsymbol{R}_i-2\boldsymbol{R}_i^T\boldsymbol{E}^{-1}(\sum_{\sigma^2_{jk}>0}\gamma_{ijk}H_{jk})\boldsymbol{M}_i+\boldsymbol{M}_i^T(\sum_{\sigma^2_{jk}>0}\gamma_{ijk}H_{jk})^T\boldsymbol{E}^{-1}(\sum_{\sigma^2_{jk}>0}\gamma_{ijk}H_{jk})\boldsymbol{M}_i \qquad \text{(S1)}\\
&=\boldsymbol{R}_i^T\boldsymbol{E}^{-1}\boldsymbol{R}_i-2\sum_{\sigma^2_{jk}>0}\gamma_{ijk}\boldsymbol{R}_i^T\boldsymbol{E}^{-1}H_{jk}\boldsymbol{M}_i+\sum_{\sigma^2_{jk}>0,\sigma^2_{jk'}>0}\gamma_{ijk}\boldsymbol{M}_i^TH_{jk}^T\boldsymbol{E}^{-1}H_{jk'}\boldsymbol{M}_i\gamma_{ijk'}
\end{aligned}$$

Let $\boldsymbol{\omega}_1=(\ldots,\boldsymbol{R}_i^T\boldsymbol{E}^{-1}H_{jk}\boldsymbol{M}_i,\ldots)=(\ldots,\frac{R_{ij}M_{ik}}{\sigma^2_{\varepsilon_j}},\ldots)$, where $\left\{(j,k):\sigma^2_{jk}>0\right\}$.
For node $j$, let $\boldsymbol{\omega}_{2j}$ be a matrix with entry $(k,k')$ as $\frac{M_{ik}M_{ik'}}{\sigma^2_{\varepsilon_j}}$, where $\left\{k,k':\sigma^2_{jk}>0,\sigma^2_{jk'}>0\right\}$.
Then

$$(S1)=\boldsymbol{R}_i^T\boldsymbol{E}^{-1}\boldsymbol{R}_i-2\gamma_i^T\boldsymbol{\omega}_1+\gamma_i^T\boldsymbol{\omega}_2\gamma_i \qquad \text{(S2)}$$

We obtain that

$$\int p(\boldsymbol{M}_i;\boldsymbol{X}_i|\gamma_i)p(\gamma_i)d\gamma_i$$
$$\propto \int exp\left(-\frac{1}{2}(\boldsymbol{R}_i^T\boldsymbol{E}^{-1}\boldsymbol{R}_i - 2\gamma_i^T\boldsymbol{\omega}_1 + \gamma_i^T(\boldsymbol{\omega}_2 + G^{-1})\gamma_i)\right)d\gamma_i$$
$$= exp\left(-\frac{1}{2}(\boldsymbol{R}_i^T\boldsymbol{E}^{-1}\boldsymbol{R}_i - \boldsymbol{\omega}_1^T(\boldsymbol{\omega}_2 + G^{-1})^{-T}\boldsymbol{\omega}_1)\right)$$
$$\times \int exp\left(\frac{1}{2}(\gamma_i - (\boldsymbol{\omega}_2 + G^{-1})^{-1}\boldsymbol{\omega}_1)^T(\boldsymbol{\omega}_2 + G^{-1})(\gamma_i - (\boldsymbol{\omega}_2 + G^{-1})^{-1}\boldsymbol{\omega}_1))\right)d\gamma_i$$
$$\propto exp\left(-\frac{1}{2}(\boldsymbol{R}_i^T\boldsymbol{E}^{-1}\boldsymbol{R}_i - \boldsymbol{\omega}_1^T(\boldsymbol{\omega}_2 + G^{-1})^{-1}\boldsymbol{\omega}_1)\right)|\boldsymbol{\omega}_2 + G^{-1}|^{1/2}$$

For node $j$, let $\boldsymbol{M}_{i,\backslash j}$ be a vector of $M_{ik}$ where $\left\{k : \sigma_{jk}^2 > 0\right\}$

$$(\boldsymbol{R}_i^T\boldsymbol{E}^{-1}\boldsymbol{R}_i - \boldsymbol{\omega}_1^T(\boldsymbol{\omega}_2 + G^{-1})^{-1}\boldsymbol{\omega}_1)_j$$
$$= \frac{R_{ij}^2}{\sigma_{\varepsilon_j}^2} - \frac{R_{ij}\boldsymbol{M}_{i,\backslash j}^T}{\sigma_{\varepsilon_j}^2}\left(\frac{\boldsymbol{M}_{i,\backslash j}\boldsymbol{M}_{i,\backslash j}^T}{\sigma_{\varepsilon_j}^2} + G_j^{-1}\right)^{-1}\frac{\boldsymbol{M}_{i,\backslash j}R_{ij}}{\sigma_{\varepsilon_j}^2}$$
$$= \frac{R_{ij}^2}{\sigma_{\varepsilon_j}^2}\left(1 - \boldsymbol{M}_{i,\backslash j}^T(\boldsymbol{M}_{i,\backslash j}\boldsymbol{M}_{i,\backslash j}^T + \sigma_{\varepsilon_j}^2G_j^{-1})^{-1}\boldsymbol{M}_{i,\backslash j}\right)$$
$$= \frac{R_{ij}^2}{\sigma_{\varepsilon_j}^2}(1 + \frac{\boldsymbol{M}_{i,\backslash j}^TG_j\boldsymbol{M}_{i,\backslash j}}{\sigma_{\varepsilon_j}^2})^{-1}$$
$$= \frac{R_{ij}^2}{\sigma_{\varepsilon_j}^2 + \sum_{k\neq j}\sigma_{jk}^2M_{ik}}$$

Therefore,

$$-2\log(\int p(\boldsymbol{M}_i;\boldsymbol{X}_i|\gamma_i)p(\gamma_i)d\gamma_i)$$
$$\propto \sum_{i=1}^n\sum_{j=1}^p\frac{R_{ij}^2}{\sigma_{\varepsilon_j}^2 + \sum_{k\neq j}\sigma_{jk}^2M_{ik}} + \sum_{i=1}^n\log(|\boldsymbol{\omega}_2 + G^{-1}|)$$
$$= \sum_{i=1}^n\sum_{j=1}^p\left(\frac{(M_{ij} - \sum_{k!=j}(\boldsymbol{\beta}_{jk}^T\boldsymbol{X}_i)M_{ik})^2}{\sum_{k\neq j}\sigma_{jk}^2M_{ik} + \sigma_{\varepsilon_j}^2} + \log(\sum_{k\neq j}\sigma_{jk}^2M_{ik}^2 + \sigma_{\varepsilon_j}^2)\right)$$

## 2 S2: ALGORITHM FOR CHECKING ACYCLIC CONSTRAINT

Algorithm 2 describes a fast and general algorithm to examine whether the acyclic constraint for a DAG is satisfied. Define $\boldsymbol{C}_j = \{k : j \leftarrow k\}$ as the ancestral set of node $j$, which includes all the direct and indirect ancestors of node $j$. For example, if there are directed edges $(j_1 \leftarrow j_2)$ and $(j_2 \leftarrow j_3)$, then $\boldsymbol{C}_{j_1} = \{j_2, j_3\}$

and $\boldsymbol{C}_{j_2} = \{j_3\}$. Node $j_3$ is an indirect ancestor of $j_1$ although they are not directly connected. The idea of the search algorithm is based on recursion. Given the current ancestral sets $\{C_j : j = 1, \cdots, p\}$, when adding a new edge $(j_1 \leftarrow j_2)$ to this set, we only need to update all the parents of $j_2$ and all the children of $j_1$. This algorithm operates iteratively to examine the cyclic constraint.

| **Algorithm 2: DAG-Checking** |
| --- |
| 1.Obtain the non-zero edge set $\boldsymbol{A}$.<br>2.Initialize $\boldsymbol{C}_j = \emptyset,\ j = 1, \cdots, p$. For $(j,k)$ belongs to $\boldsymbol{A}$, do<br>a.Update $\boldsymbol{C}_j = \boldsymbol{C}_j \cup k$.<br>b.Update the parent of $j$, $\boldsymbol{C}_j = \boldsymbol{C}_j \cup \boldsymbol{C}_k$.<br>c.Update the child of $j$, $\boldsymbol{C}_{j_1} = \boldsymbol{C}_{j_1} \cup C_j$ for all $j_1$ where $j \in \boldsymbol{C}_{j_1}$.<br>3.$\boldsymbol{A}$ is a DAG if $(j,j) \notin \boldsymbol{C}_j$ for all $j = 1, \cdots, p$; otherwise, $\boldsymbol{A}$ is not a DAG. |

## 3 S3: PROOF OF THEOREM 1

We have

$$\sum_{j=1}^{p}\left[\frac{(M_j - \sum_{k\neq j}(\boldsymbol{\beta}_{0jk}^T X)M_k)^2}{\sum_{k\neq j}\sigma_{0jk}^2 M_k^2 + \sigma_{\varepsilon_j}^2} + \log(\sum_{k\neq j}\sigma_{0jk}^2 M_k^2 + \sigma_{0\varepsilon_j}^2)\right]$$

$$= \sum_{j=1}^{p}\left[\frac{(M_j - \sum_{k\neq j}(\boldsymbol{\beta}_{jk}^T X)M_k)^2}{\sum_{k\neq j}\sigma_{jk}^2 M_k^2 + \sigma_{\varepsilon_j}^2} + \log(\sum_{k\neq j}\sigma_{jk}^2 M_k^2 + \sigma_{\varepsilon_j}^2)\right]. \quad (1)$$

We wish to show $(B_0, \Sigma_0, \theta_0) = (B, \Sigma, \theta)$. First, we start with any terminal node in the DAG defined by $(B, \Sigma, \theta)$ (it always exists) so WLOG, we suppose $p$ to be such a node so $\boldsymbol{\beta}_{jp} = 0$ and $\sigma_{jp}^2 = 0$ for any $j \neq p$. In the left-hand side of (1), the term containing $M_p$ is

$$\frac{(M_p - \sum_{k\in pa(p)}(\boldsymbol{\beta}_{pk}^T X)M_k)^2}{\sum_{k\in pa(p)}\sigma_{pk}^2 M_k^2 + \sigma_{\varepsilon_p}^2}$$

which is a quadratic function of $M_p$. On the right-hand side of (1), the terms containing $M_p$ are

$$\frac{(M_p - \sum_{k\in pa_0(p)}(\boldsymbol{\beta}_{0pk}^T X)M_k)^2}{\sum_{k\in pa_0(p)}\sigma_{0pk}^2 M_k^2 + \sigma_{0\varepsilon_p}^2}$$

$$+ \sum_{j\in ch_0(p)}\left[\frac{(M_j - \sum_{k\neq j,k\neq p}(\boldsymbol{\beta}_{0jk}^T X)M_k - (\boldsymbol{\beta}_{0jp}^T X)^T M_p)^2}{\sum_{k\neq j,k\neq p}\sigma_{0jk}^2 M_k^2 + \sigma_{0jp}^2 M_p^2 + \sigma_{0\varepsilon_j}^2} + \log(\sum_{k\neq j,k\neq p}\sigma_{0jk}^2 M_k^2 + \sigma_{0jp}^2 M_p^2 + \sigma_{0\varepsilon_j}^2)\right],$$

where $pa(p)$ denotes the parental nodes for node $p$ in the DAG and $pa_0(p)$ and $ch_0(p)$ are the parental and child nodes in the true DAG, respectively. Thus, we have

$$\frac{(M_p - \sum_{k\in pa(p)}(\boldsymbol{\beta}_{pk}^T X)M_k)^2}{\sum_{k\in pa(p)}\sigma_{pk}^2 M_k^2 + \sigma_{\varepsilon_p}^2} - \frac{(M_p - \sum_{k\in pa_0(p)}(\boldsymbol{\beta}_{0pk}^T X)M_k)^2}{\sum_{k\in pa_0(p)}\sigma_{0pk}^2 M_k^2 + \sigma_{0\varepsilon_p}^2}$$

$$= \sum_{j \in ch_0(p)} \left[ \frac{(M_j - \sum_{k \neq j, k \neq p} (\boldsymbol{\beta}_{0jk}^T X) M_k - (\boldsymbol{\beta}_{0jp}^T X)^T M_p)^2}{\sum_{k \neq j, k \neq p} \sigma_{0jk}^2 M_k + \sigma_{0jp}^2 M_p^2 + \sigma_{0\varepsilon_j^2}} + \log( \sum_{k \neq j, k \neq p} \sigma_{0jk}^2 M_k + \sigma_{0jp}^2 M_p + \sigma_{0\varepsilon_j}^2 ) \right] + c \tag{2}$$

for some $c$ only depending on $M_j$'s for $j \neq p$.

We first show that for node $p$, $ch_0(p)$ should be empty. Suppose that $\chi_0(p)$ is non-empty. Clearly, $\sigma_{jp}^2 = 0$ for all $j \in ch(p)$ by examining the behavior when $M_p \to \infty$ in (2). Hence, $\boldsymbol{\beta}_{0jp} \neq 0$ by the definition of $ch_0(p)$. We then obtain

$$\frac{(M_p - \sum_{k \in pa(p)} (\boldsymbol{\beta}_{pk}^T X) M_k)^2}{\sum_{k \in pa(p)} \sigma_{pk}^2 M_k^2 + \sigma_{\varepsilon_p}^2} - \frac{(M_p - \sum_{k \in pa_0(p)} (\boldsymbol{\beta}_{0pk}^T X) M_k)^2}{\sum_{k \in pa_0(p)} \sigma_{0pk}^2 M_k^2 + \sigma_{0\varepsilon_p}^2}$$

$$= \sum_{j \in ch_0(p)} \left[ \frac{(M_j - \sum_{k \neq j, k \neq p} (\boldsymbol{\beta}_{0jk}^T X) M_k - (\boldsymbol{\beta}_{0jp}^T X)^T M_p)^2}{\sum_{k \neq j, k \neq p} \sigma_{0jk}^2 M_k^2 + \sigma_{0\varepsilon_j^2}} + \log( \sum_{k \neq j, k \neq p} \sigma_{0jk}^2 M_k^2 + \sigma_{0\varepsilon_j}^2 ) \right] + c.$$

We further compare the coefficients of $M_p^2$ and $M_p$ to obtain

$$\frac{1}{\sum_{k \in pa(p)} \sigma_{pk}^2 M_k^2 + \sigma_{\varepsilon_p}^2} = \frac{1}{\sum_{k \in pa_0(p)} \sigma_{0pk}^2 M_k^2 + \sigma_{0\varepsilon_p}^2} + \sum_{j \in ch_0(p)} \left[ \frac{((\boldsymbol{\beta}_{0jp}^T X)^T)^2}{\sum_{k \neq j, k \neq p} \sigma_{0jk}^2 M_k^2 + \sigma_{0\varepsilon_j^2}} \right] \tag{3}$$

and

$$\frac{\sum_{k \in pa(p)} (\boldsymbol{\beta}_{pk}^T X) M_k}{\sum_{k \in pa(p)} \sigma_{pk}^2 M_k^2 + \sigma_{\varepsilon_p}^2} = \frac{\sum_{k \in pa_0(p)} (\boldsymbol{\beta}_{0pk}^T X) M_k}{\sum_{k \in pa_0(p)} \sigma_{0pk}^2 M_k^2 + \sigma_{0\varepsilon_p}^2} + \sum_{j \in ch_0(p)} \frac{(M_j - \sum_{k \neq j, k \neq p} (\boldsymbol{\beta}_{0jk}^T X) M_k)(\boldsymbol{\beta}_{0jp}^T X)}{\sum_{k \neq j, k \neq p} \sigma_{0jk}^2 M_k^2 + \sigma_{0\varepsilon_j^2}}. \tag{4}$$

In (3), if there exists some $j \in ch_0(p)$ and some $k$ such that $k \neq j, p$ and $k \notin pa_0(p) \cup pa(p)$ and $\sigma_{0jk}^2 \neq 0$, then we let $M_j = 0$ for any $j \neq k$ to obtain

$$0 = \sum_{j \in ch_0(p)} \frac{(\boldsymbol{\beta}_{0jp}^T X)^2}{\sigma_{0jk}^2 M_k^2 + \sigma_{0\varepsilon_j}^2}.$$

Thus, $\boldsymbol{\beta}_{0jp}^T X = 0$ so $\boldsymbol{\beta}_{0jp} = 0$. This gives a contradiction. Therefore, (3) can be rewritten as

$$\frac{1}{\sum_{k \in pa(p)} \sigma_{pk}^2 M_k^2 + \sigma_{\varepsilon_p}^2} = \frac{1}{\sum_{k \in pa_0(p)} \sigma_{0pk}^2 M_k^2 + \sigma_{0\varepsilon_p}^2} + \sum_{j \in ch_0(p)} \left[ \frac{((\boldsymbol{\beta}_{0jp}^T X)^T)^2}{\sum_{k \in pa_0(p) \cup pa(p)} \sigma_{0jk}^2 M_k^2 + \sigma_{0\varepsilon_j^2}} \right] \tag{5}.$$

If there exits some $k \in pa(p)$ but $k \notin pa_0(p)$, we let $M_j = 0$ for any $j \neq k$ in (5) to obtain

$$\frac{1}{\sigma_{pk}^2 M_k^2 + \sigma_{\varepsilon_p}^2} = \sum_{j \in ch_0(p)} \left[ \frac{((\boldsymbol{\beta}_{0jp}^T X)^T)^2}{\sigma_{0jk}^2 M_k^2 + \sigma_{0\varepsilon_j^2}} \right].$$

This implies that for $j \in ch_0(p)$, if $\sigma_{0jk} = 0$, then $\beta_{0jp}^T X = 0$ so $\beta_{0jp} = 0$; if $\sigma_{0jk} \neq 0$, then $(\beta_{0jp}^T X)^2/\sigma_{0jk}^2 = 1/\sigma_{pk}^2$. Therefore, $\sum_{j \in ch_0(p)} (\beta_{0jp}^T X)^2$ is a constant. According to the heterogeneity assumption, we obtain another contradiction. We conclude $pa(p) \subset pa_0(p)$. Similarly, if there exists some $k \in pa_0(p)$ but $k \notin pa(p)$, we let $M_j = 0$ for any $j \neq k$ to obtain

$$0 = \frac{1}{\sigma_{0pk}^2 M_k^2 + \sigma_{0\varepsilon_p}^2} + \sum_{j \in ch_0(p)} \left[ \frac{((\boldsymbol{\beta}_{0jp}^T X)^T)^2}{\sigma_{0jk}^2 M_k^2 + \sigma_{0\varepsilon_j^2}} \right],$$

which is impossible. Thus, $pa_0(p) = pa(p)$ so (3) and (4) become

$$\frac{(\sum_{k \in pa_0(p)} (\boldsymbol{\beta}_{pk}^T X) M_k)^2}{\sum_{k \in pa_0(p)} \sigma_{pk}^2 M_k^2 + \sigma_{\varepsilon_p}^2} = \frac{(\sum_{k \in pa_0(p)} (\boldsymbol{\beta}_{0pk}^T X) M_k)^2}{\sum_{k \in pa_0(p)} \sigma_{0pk}^2 M_k^2 + \sigma_{0\varepsilon_p}^2} + \sum_{j \in ch_0(p)} \left[ \frac{((\boldsymbol{\beta}_{0jp}^T X)^T)^2}{\sum_{k \in pa_0(p)} \sigma_{0jk}^2 M_k^2 + \sigma_{0\varepsilon_j^2}} \right] \quad (6)$$

and

$$\frac{\sum_{k \in pa_0(p)} (\boldsymbol{\beta}_{pk}^T X) M_k}{\sum_{k \in pa_0(p)} \sigma_{pk}^2 M_k^2 + \sigma_{\varepsilon_p}^2} = \frac{\sum_{k \in pa_0(p)} (\boldsymbol{\beta}_{0pk}^T X) M_k}{\sum_{k \in pa_0(p)} \sigma_{0pk}^2 M_k^2 + \sigma_{0\varepsilon_p}^2} + \sum_{j \in ch_0(p)} \frac{(M_j - \sum_{k \neq j, k \neq p} (\boldsymbol{\beta}_{0jk}^T X) M_k)(\boldsymbol{\beta}_{0jp}^T X)}{\sum_{k \in pa_0(p)} \sigma_{0jk}^2 M_k^2 + \sigma_{0\varepsilon_j^2}}. \quad (7)$$

We examine the second term in the right-hand side of (7). In particular, there always exits a $j$ in $ch_0(p)$ which cannot be the parental node for any other nodes in $ch_0(p)$. For such $j$, the coefficient of $M_j$ in this term is $\boldsymbol{\beta}_{0jp}^T X / [\sum_{k \in pa_0(p)} \sigma_{0jk}^2 M_k^2 + \sigma_{0\varepsilon_j}^2]$ which is not zero. Since such $j$ cannot be in $pa_0(p)$, we have the contradiction. In other words, $ch_0(p)$ must empty.

Since $ch_0(p)$ is empty, (2) becomes

$$\frac{(M_p - \sum_{k \in pa_0(p)} (\boldsymbol{\beta}_{pk}^T X) M_k)^2}{\sum_{k \in pa_0(p)} \sigma_{pk}^2 M_k^2 + \sigma_{\varepsilon_p}^2} = \frac{(M_p - \sum_{k \in pa_0(p)} (\boldsymbol{\beta}_{0pk}^T X) M_k)^2}{\sum_{k \in pa_0(p)} \sigma_{0pk}^2 M_k^2 + \sigma_{0\varepsilon_p}^2}$$

so after comparing the coefficients of $M_p$ and $M_p^2$, along with the same arguments as before, we obtain $pa_0(p) = pa_0(p)$ and moreover,

$$\frac{(\sum_{k \in pa_0(p)} (\boldsymbol{\beta}_{pk}^T X) M_k)^2}{\sum_{k \in pa_0(p)} \sigma_{pk}^2 M_k^2 + \sigma_{\varepsilon_p}^2} = \frac{(\sum_{k \in pa_0(p)} (\boldsymbol{\beta}_{0pk}^T X) M_k)^2}{\sum_{k \in pa_0(p)} \sigma_{0pk}^2 M_k^2 + \sigma_{0\varepsilon_p}^2} \quad (8)$$

and

$$\frac{\sum_{k \in pa_0(p)} (\boldsymbol{\beta}_{pk}^T X) M_k}{\sum_{k \in pa_0(p)} \sigma_{pk}^2 M_k^2 + \sigma_{\varepsilon_p}^2} = \frac{\sum_{k \in pa_0(p)} (\boldsymbol{\beta}_{0pk}^T X) M_k}{\sum_{k \in pa_0(p)} \sigma_{0pk}^2 M_k^2 + \sigma_{0\varepsilon_p}^2}. \quad (9)$$

Then for any $k \in pa_0(p)$, we let $M_{k'} = 0$ for $k' \neq k$ in (8) and (9) to obtain

$$\frac{(\boldsymbol{\beta}_{pk}^T X)^2 M_k^2}{\sigma_{pk}^2 M_k^2 + \sigma_{\varepsilon_p}^2} = \frac{(\boldsymbol{\beta}_{0pk}^T X)^2 M_k^2}{\sigma_{0pk}^2 M_k^2 + \sigma_{0\varepsilon_p}^2}, \quad \frac{(\boldsymbol{\beta}_{pk}^T X) M_k}{\sigma_{pk}^2 M_k^2 + \sigma_{\varepsilon_p}^2} = \frac{(\boldsymbol{\beta}_{0pk}^T X) M_k}{\sigma_{0pk}^2 M_k^2 + \sigma_{0\varepsilon_p}^2}.$$

Immediately, we have $\boldsymbol{\beta}_{0pk}^T X = \boldsymbol{\beta}_{pk}^T X$ so $\boldsymbol{\beta}_{0pk} = \boldsymbol{\beta}_{pk}$. Thus, (9) gives

$$\sum_{k \in pa_0(p)} \sigma_{pk}^2 M_k^2 + \sigma_{\varepsilon_p}^2 = \sum_{k \in pa_0(p)} \sigma_{0pk}^2 M_k^2 + \sigma_{0\varepsilon_p}^2$$

so it is clear that $\sigma_{pk}^2 = \sigma_{0pk}^2$ and $\sigma_{\varepsilon_p}^2 = \sigma_{0\varepsilon_p}^2$.

In other words, we have shown that if the node $p$ is one terminal node in the DAG given by $(B, \Sigma)$, it is also a terminal one in the true DAG. Furthermore, all $\boldsymbol{\beta}_{pk}$ and $\sigma_{pk}^2$ as well as $\sigma_{\varepsilon_p}^2$ are the same between the two DAGs. Thus, after removing this node $p$ from both DAGs, the likelihood equality in (1) reduces to

$$\sum_{j=1}^{p-1} \left[ \frac{(M_j - \sum_{k \neq j} (\boldsymbol{\beta}_{jk}^T X) M_k)^2}{\sum_{k \neq j} \sigma_{jk}^2 M_k^2 + \sigma_{0\varepsilon_j}^2} + \log(\sum_{k \neq j} \sigma_{jk}^2 M_k^2 + \sigma_{\varepsilon_j}^2) \right]$$

$$= \sum_{j=1}^{p-1} \left[ \frac{(M_j - \sum_{k \neq j} (\boldsymbol{\beta}_{0jk}^T X) M_k)^2}{\sum_{k \neq j} \sigma_{0jk}^2 M_k^2 + \sigma_{0\varepsilon_j}^2} + \log(\sum_{k \neq j} \sigma_{0jk}^2 M_k^2 + \sigma_{0\varepsilon_j}^2) \right].$$

We then continue the same arguments till exhausting all the nodes in the true DAGs (equivalently, by induction on the DAG size). The identifiability thus holds.

# 4 S4: ADDITIONAL RESULTS OF BRAIN ATROPHY DEPENDENCE NETWORK ANALYSIS

**Table S1.** Summary of five subcortical gray matter connections identified by DAG-MM2 in PREDICT-HD study.

| Edge (parent ⇒ child) | Intercept * | $L_2$ norm # | $\sigma^2$ $ |
|---|---|---|---|
| L.Amygdala⇒L.Cerebellum Cortex | 0.469 | 0.576 | 0.743 |
| R.Pallidum⇒L.Thalamus | 0.449 | 0.553 | 0.907 |
| R.Pallidum⇒L.Putamen | 0.385 | 0.435 | 0.729 |
| L.Thalamus⇒R.Accumbens | 0.813 | 0.904 | 0.791 |
| R.Pallidum ⇒R.VentralDC | 0.344 | 0.450 | 0.795 |

*: Estimated intercept ; #: $L_2$ norm of all the estimated covariate coefficients for the edge connection; $: Variance of random effects for each connection.

**Table S2.** Summary of 58 cortical gray matter connections identified by DAG-MM in PREDICT-HD study.

| Edge (parent⇒child) | Intercept * | $L_2$ norm # | $\sigma^2$ $ | Edge (parent⇒child) | Intercept * | $L_2$ norm # | $\sigma^2$ $ |
|---|---|---|---|---|---|---|---|
| L.middle temporal ⇒ L.bankssts | 0.553 | 0.694 | 0.735 | R.superior temporal⇒L.transverse temporal | 0.143 | 0.511 | 0.507 |
| R.bankssts⇒L.bankssts | 0.364 | 0.475 | 0.510 | L.bankssts⇒L.insula | 0.473 | 0.556 | 0.861 |
| L.fusiform⇒L.caudal anterior cingulate | 0.267 | 0.396 | 0.476 | R.middle temporal⇒R.bankssts | 0.762 | 0.777 | 0.430 |
| L.rostral middle frontal⇒L.caudal anterior cingulate | 0.220 | 0.388 | 0.473 | R.posterior cingulate⇒R.caudal anterior cingulate | 0.855 | 0.954 | 0.668 |
| L.postcentral⇒L.caudal middle frontal | 0.523 | 0.584 | 0.447 | R.superior temporal⇒R.cuneus | 0.298 | 0.321 | 0.453 |
| L.lingual⇒L.cuneus | 0.641 | 0.669 | 0.694 | R.middle temporal⇒R.entorhinal | 0.642 | 0.724 | 0.517 |
| R.inferior temporal ⇒L.entorhinal | 0.759 | 0.895 | 0.501 | L.parsopercularis⇒R.inferior parietal | 0.666 | 0.732 | 0.683 |
| R.lingual⇒L.fusiform | 0.426 | 0.484 | 0.441 | L.isthmus cingulate⇒R.isthmus cingulate | 0.485 | 0.547 | 0.444 |
| R.superior temporal⇒L.fusiform | 0.379 | 0.421 | 0.417 | R.inferior parietal⇒R.isthmus cingulate | 0.362 | 0.587 | 0.535 |
| L.caudal middle frontal⇒L.inferior parietal | 0.759 | 0.854 | 0.717 | R.fusiform⇒R.lateral occipital | 0.503 | 0.677 | 0.520 |
| L.middle temporal⇒L.inferior temporal | 0.858 | 0.898 | 0.423 | R.lingual⇒R.lateral occipital | 0.621 | 0.647 | 0.442 |
| L.precuneus⇒L.isthmus cingulate | 0.263 | 0.445 | 0.609 | R.inferior temporal ⇒R.lateral orbitofrontal | 0.605 | 0.631 | 0.472 |
| L.insula⇒L.lateral orbitofrontal | 0.560 | 0.643 | 0.556 | R.middle temporal⇒R.lateral orbitofrontal | -0.386 | 0.732 | 0.531 |
| L.fusiform⇒L.lingual | 0.741 | 0.832 | 0.783 | R.inferior parietal⇒R.lingual | 0.660 | 0.737 | 0.709 |
| L.middle temporal⇒L.lingual | 0.020 | 0.421 | 0.710 | R.superior temporal⇒R.lingual | 0.183 | 0.487 | 0.423 |
| L.middle temporal⇒L.medial orbitofrontal | 0.366 | 0.505 | 0.556 | R.bankssts⇒R.parahippocampal | -0.044 | 0.282 | 0.451 |
| L.fusiform⇒L.middle temporal | 0.792 | 0.853 | 0.390 | R.fusiform⇒R.parahippocampal | 0.857 | 0.959 | 0.438 |
| R.superior temporal⇒L.parahippocampal | 0.526 | 0.589 | 0.693 | L.precentral⇒R.paracentral | 0.863 | 0.898 | 0.596 |
| R.middle temporal⇒L.parsopercularis | 0.736 | 0.846 | 0.499 | L.rostral middle frontal⇒R.parsopercularis | 0.442 | 0.801 | 0.665 |
| L.temporal pole⇒L.parsorbitalis | 0.126 | 0.310 | 0.445 | L.inferior temporal⇒R.parsorbitalis | 0.002 | 0.533 | 0.478 |
| L.middle temporal⇒ L.parstriangularis | 0.606 | 0.734 | 0.675 | R.parahippocampal⇒R.pericalcarine | 0.171 | 0.272 | 0.513 |
| L.caudal middle frontal⇒L.posterior cingulate | 0.339 | 0.434 | 0.895 | R.lingual⇒R.postcentral | 0.447 | 0.593 | 0.490 |
| R.supramarginal⇒L.precuneus | 0.903 | 0.964 | 0.623 | L.posterior cingulate⇒R.posterior cingulate | 0.454 | 0.516 | 0.640 |
| R.inferior temporal⇒L.rostral anterior cingulate | 0.567 | 0.661 | 0.391 | R.inferior temporal⇒R.rostral anterior cingulate | 0.571 | 0.702 | 0.469 |
| L.caudal middle frontal⇒L.rostral middle frontal | 0.916 | 0.979 | 0.602 | R.inferior parietal⇒R.superior parietal | 0.869 | 0.883 | 0.566 |
| R.superior temporal⇒L.superior temporal | 0.907 | 0.925 | 0.377 | R.inferior parietal⇒R.supramarginal | 0.793 | 0.806 | 0.413 |
| R.supramarginal⇒L.supramarginal | 0.819 | 0.857 | 0.409 | L.transverse temporal⇒R.temporal pole | 0.287 | 0.401 | 0.587 |
| R.entorhinal⇒L.temporal pole | 0.451 | 0.625 | 0.594 | L.pericalcarine⇒R.transverse temporal | 0.402 | 0.461 | 0.551 |
| R.bankssts⇒L.transverse temporal | 0.254 | 0.402 | 0.577 | R.fusiform⇒R.insula | 0.544 | 0.597 | 0.507 |

* Intercept: Estimated intercept ; # $L_2$ norm: L2 norm of all the estimated covariate coefficients for the connection; $ $\sigma^2$ : Variance of random effects.

**Table S3.** Module-wise white matter connectivity network measured by DTI technology and probabilistic tractography (TRACK-ON study).

| Modular connection | Number of links# | Estimates norm * |
|---|---|---|
| **Intramodular connection** | | |
| R.fronto-cingulate/R.fronto-cingulate | 2 | 0.348 |
| R.motor-occipital-parietal/R.motor-occipital-parietal | 2 | 0.320 |
| **Intrahemispheric connection** | | |
| R.fronto-cingulate/R.motor-occipital-parietal | 2 | 0.177 |
| L.fronto-cingulate/L.temporal | 1 | 0.179 |
| L.fronto-cingulate/L.motor-occipital-parietal | 1 | 0.154 |
| L.temporal/L.motor-occipital-parietal | 1 | 0.210 |
| **Interhemispheric connection** | | |
| L.motor-occipital-parietal/R.motor-occipital-parietal | 7 | 0.580 |
| R.fronto-cingulate/L.motor-occipital-parietal | 5 | 0.699 |
| L.fronto-cingulate/R.fronto-cingulate | 2 | 0.281 |
| L.fronto-cingulate/R.motor-occipital-parietal | 2 | 0.312 |
| L.temporal/R.motor-occipital-parietal | 2 | 0.365 |
| L.fronto-fingulate/R.temporal | 1 | 0.180 |
| R.fronto-cingulate/L.temporal | 1 | 0.238 |
| R.temporal/L.motor-occipital-parietal | 1 | 0.228 |

# Number of links: Number of cortical links in the modular connection ; * Estimates norm: L2 norm of the standardized time fixed effect coefficients estimated by linear mixed effects model for the cortical links in the modular connection.

**Table S4.** DAG-MM2 estimated connection strength associated with covariates in the gray matter cortical network (PREDICT-HD study)

| Covariate | Subcortical* | Cortical* |
|---|---|---|
| Intercept | 1.161 | 4.365 |
| CAP median | 0.247 | 1.338 |
| CAP high | 0.363 | 1.589 |
| Older | 0.136 | 1.031 |
| Sex | 0.413 | 0.913 |
| TFC | 0.197 | 0.418 |
| TMS | 0.145 | 0.449 |
| SDMT | 0.236 | 0.532 |

* $L_2$ norm of all the estimated connection coefficients for each covariate.

**Figure S1.** Estimated node-wise cortical thickness atrophy dependence networks. Yellow nodes: connected nodes. Black nodes: singular nodes.

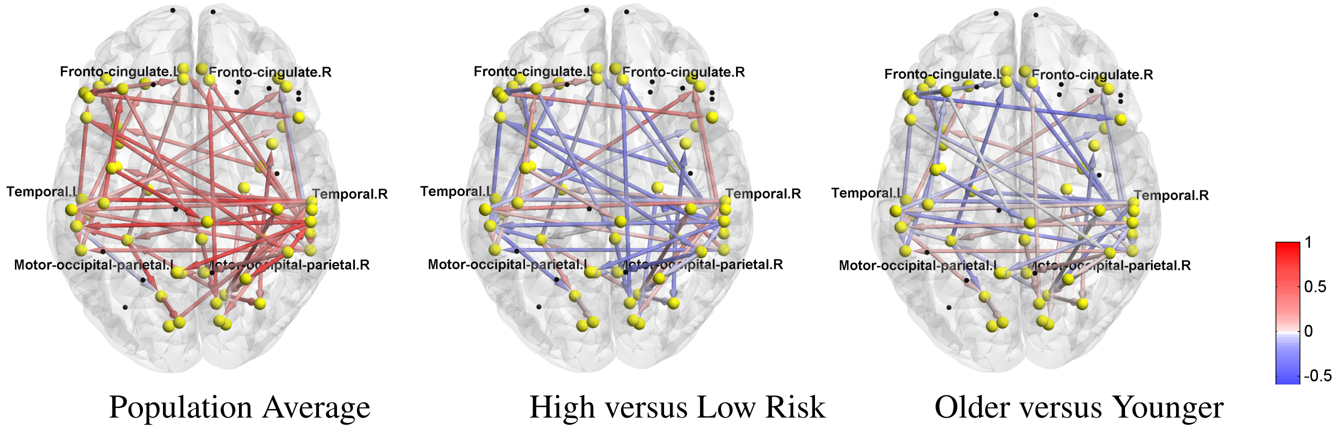

**Figure S2.** Estimated variation of node-wise cortical thickness atrophy dependence networks. Yellow nodes: connected nodes; Black nodes: singular nodes; Edge color represents the magnitude of the variation of heterogeneous effects (standard deviation $\sigma_{jk}$).

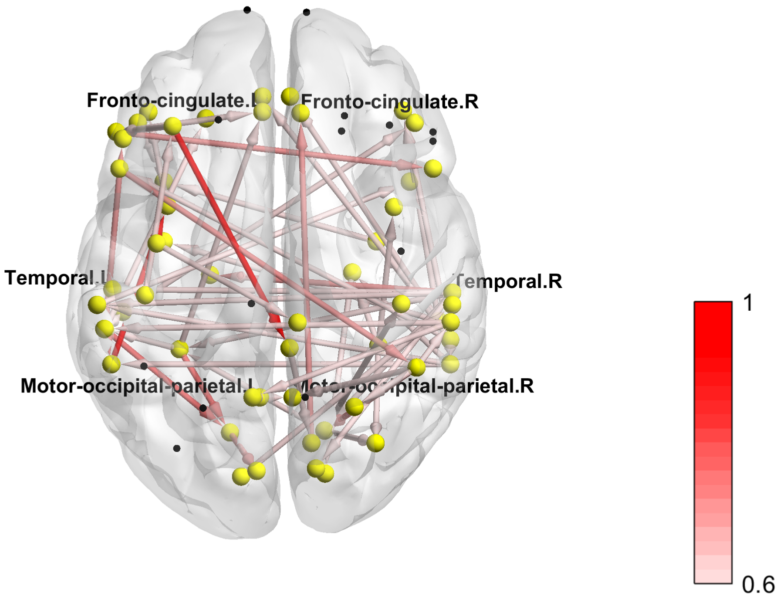

Supplement: Supplementary file 1 [file Data_Sheet_1.PDF]
